# Supplementary material for: Secondary through-space interactions facilitated single-molecule white-light emission from clusteroluminogens
Source: Nat Commun. 2022 Jun 17;13:3492. doi: 10.1038/s41467-022-31184-9 (PMC9205862; doi:10.1038/s41467-022-31184-9)
Supplement: Supplementary file 1 — Supplementary Information [file 41467_2022_31184_MOESM1_ESM.pdf]

Supplementary Information

Secondary through-space interactions facilitated single-molecule white-light emission from clusteroluminogens

Jianyu Zhang[1], Parvej Alam[1], Siwei Zhang[1], Hanchen Shen[1], Lianrui Hu[1], Herman H. Y. Sung[1], Ian D. Williams[1], Jianwei Sun[1], Jacky W. Y. Lam[1]*, Haoke Zhang[2,3,4]* & Ben Zhong Tang[1,4,5]*

[1]Department of Chemistry, Hong Kong Branch of Chinese National Engineering Research Center for Tissue Restoration and Reconstruction, and Guangdong-Hong Kong-Macau Joint Laboratory of Optoelectronic and Magnetic Functional Materials, The Hong Kong University of Science and Technology, Clear Water Bay, Kowloon, Hong Kong 999077, China

[2]MOE Key Laboratory of Macromolecular Synthesis and Functionalization, Department of Polymer Science and Engineering, Zhejiang University, Hangzhou 310027, China

[3]ZJU-Hangzhou Global Scientific and Technological Innovation Center, Hangzhou 311215, China

[4]Guangdong Provincial Key Laboratory of Luminescence from Molecular Aggregates, South China University of Technology, Guangzhou 510640, China

[5]School of Science and Engineering, Shenzhen Institute of Aggregate Science and Technology, The Chinese University of Hong Kong, Shenzhen, Guangdong 518172, China

[#]These authors contributed equally: Jianyu Zhang, Parvej Alam, and Siwei Zhang.

*Corresponding emails: tangbenz@cuhk.edu.cn (Ben Zhong Tang); zhanghaoke@zju.edu.cn (Haoke Zhang); chjacky@ust.hk (Jacky W. Y. Lam)

Table of Contents

Synthesis 4
Supplementary Figure 1. Synthetic routes to TPMI-Cl, TPMI-Br, and TPMI-I 4
N-tritylethane-1,2-diamine (TPM-NH_2) 4
(E)-2-[(4-chlorobenzylidene)amino]-N-tritylethan-1-amine (TPMI-Cl) 4
(E)-2-[(4-bromobenzylidene)amino]-N-tritylethan-1-amine (TPMI-Br) 4
(E)-2-[(4-iodobenzylidene)amino]-N-tritylethan-1-amine (TPMI-I) 4
Recrystallization protocol 5
Supplementary Figure 2. Synthetic routes to model compound Me-TPMA and Me-PMI-X 5
N-methyl-1,1,1-triphenylmethanamine (Me-TPMA) 5
(E)-1-(4-chlorophenyl)-N-methylmethanimine (Me-PMI-Cl) 5
(E)-1-(4-bromophenyl)-N-methylmethanimine (Me-PMI-Br) 6
(E)-1-(4-iodophenyl)-N-methylmethanimine (Me-PMI-I) 6
Nuclear Magnetic Resonance Spectra 6
Supplementary Figure 3. ^{1}H and ^{13}C NMR spectra of TPM-NH_2 in $CDCl_3$. 6
Supplementary Figure 4. ^{1}H and ^{13}C NMR spectra of TPMI-Cl in $CDCl_3$. 7
Supplementary Figure 5. ^{1}H and ^{13}C NMR spectra of TPMI-Br in $CDCl_3$. 7
Supplementary Figure 6. ^{1}H and ^{13}C NMR spectra of TPMI-I in $CDCl_3$. 8
Supplementary Figure 7. ^{1}H and ^{13}C NMR spectra of Me-TPMA in $CDCl_3$. 8
Supplementary Figure 8. ^{1}H and ^{13}C NMR spectra of Me-PMI-Cl in $CDCl_3$. 9
Supplementary Figure 9. ^{1}H and ^{13}C NMR spectra of Me-PMI-Br in $CDCl_3$. 9
Supplementary Figure 10. ^{1}H and ^{13}C NMR spectra of Me-PMI-I in $CDCl_3$. 10
High-Resolution Mass Spectrometry 10
Supplementary Figure 11. High-resolution mass spectrum of TPM-NH_2. 10
Supplementary Figure 12. High-resolution mass spectrum of TPMI-Cl. 11
Supplementary Figure 13. High-resolution mass spectrum of TPMI-Br. 11
Supplementary Figure 14. High-resolution mass spectrum of TPMI-I. 12
Supplementary Figure 15. High-resolution mass spectrum of Me-TPMA. 12
Supplementary Figure 16. High-resolution mass spectrum of Me-PMI-Cl. 13
Supplementary Figure 17. High-resolution mass spectrum of Me-PMI-Br. 13
Supplementary Figure 18. High-resolution mass spectrum of Me-PMI-I. 14
High-Performance Liquid Chromatography 14
Supplementary Figure 19. High-performance liquid chromatogram of the prepared samples 14
Other Characterizations 15
Supplementary Figure 20. Photos of THF solution, aggregate and crystal of TPMI-Cl, TPMI-Br, and TPMI-I 15
Supplementary Figure 21. The size distribution of TPMI-Cl, TPMI-Br, and TPMI-I in THF/water mixtures 15
Supplementary Figure 22. The Commission Internationale de L'Eclairage chromaticity coordinate and absolute quantum yield of TPMI-Cl, TPMI-Br, and TPMI-I in the crystalline state 16
Supplementary Figure 23. Excitation spectra of TPMI-Cl and TPMI-I 16
Supplementary Figure 24. Powder X-ray diffraction patterns and normalized photoluminescence spectra of TPMI-Cl, TPMI-Br, and TPMI-I in the amorphous state 17
Supplementary Figure 25. Normalized steady and delayed photoluminescence spectra of TPMI-Cl, TPMI-Br, and TPMI-I in the crystalline state at 77 K 17
Supplementary Figure 26. Normalized absorption spectra of *N*-methyl-triphenylmethanamine (Me-TPMA) in THF solution. 18
Supplementary Figure 27. Normalized absorption and photoluminescence spectra of halogen-substituted *N*-methyl-phenylmethanimine (Me-PMI-X, X = Cl, Br, and I) in THF solution 18
Supplementary Figure 28. Normalized photoluminescence and excitation spectra of the Me-TPMA/Me-PMI-Cl and Me-TPMA/Me-PMI-I mixture with molar ratio of 1:1 in the solid state. 19
Supplementary Table 1. Summary of crystallographic data and structural refinement of the synthesized compounds TPMI-X (X = Cl, Br and I) 20
Supplementary Figure 29. Crystal packing diagram of TPMI-Cl. 21
Supplementary Figure 30. Crystal packing diagram of TPMI-Br. 21
Supplementary Figure 31. Crystal packing diagram of TPMI-I. 22
Supplementary Figure 32. Hirshfeld surface analysis and decomposed fingerprint plots TPMI-X (X = Cl, Br and I)23
Supplementary Figure 33. Natural transition orbitals of TPMI-Cl. 24
Supplementary Figure 34. Natural transition orbitals of TPMI-I 24

Supplementary Table 2. Theoretically calculated N_1–N_2 distance and dihedral angle of $\angle N_1$-C_1-C_2-N_2 of TPMI-X. 25
Supplementary Figure 35. Spin density maps of the triplet state of TPMI-X 25
Supplementary Figure 36. Photoluminescence spectra and time-resolved photoluminescence decay curves of Me-PMI-X (X = Cl, Br, and I) in THF solution at 77 K 25
Supplementary Figure 37. Energy diagram and SOC values of TPMI-X in the isolated phase and crystalline phases 26
Supplementary Figure 38. Setup of QM/MM model for theoretical calculation of crystalline phase......................... 26
Supplementary Figure 39. Overlap of the optimized ground-state and singlet TSCT geometries of TPMI-Cl and TPMI-I in the isolated phase and crystalline phase. 27
Supplementary Figure 40. Photo of fabricated white-light emitters under the optical microscope 27
Supplementary Figure 41. The PL spectra and Commission Internationale de L'Eclairage chromaticity coordinate of a white-light emitter fabricated by TPMI-Cl 28
Supplementary Figure 42. The PL spectra and Commission Internationale de L'Eclairage chromaticity coordinate of a white-light emitter fabricated by TPMI-I.. 28
Cartesian Coordinates of the Optimized Molecular Geometry 28

Synthesis

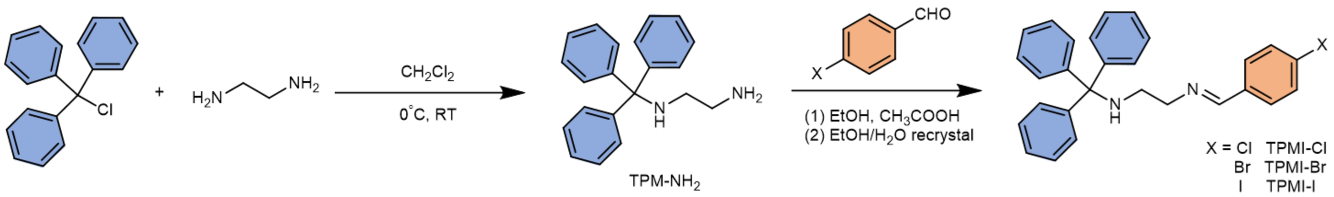

Supplementary Figure 1. Synthetic routes to TPMI-Cl, TPMI-Br, and TPMI-I.

N-tritylethane-1,2-diamine (TPM-NH₂)

7.48 mL ethylenediamine (112 mmol, 6.74 g) was added into a 250 mL two-necked flask with 70 mL dichloromethane (DCM) and cooled in ice water to keep the temperature at 0 °C. 3.90 g triphenylmethyl chloride (14 mmol) dissolved in 20 mL DCM was added into the flask drop by drop for 20 mins. The mixture was stirred at 0 °C for 2 h and at room temperature for another 7 h under nitrogen gas (Supplementary Scheme 1). Then the reaction mixture was washed with saturated NaHCO3 solution three times. The organic layer was separated and dried with enough anhydrous sodium sulfate. After filtration, the filtrate was evaporated under reduced pressure, and the crude product was purified on a silica gel column using DCM/MeOH mixture (10/1, v/v) as the eluent. 3.06 g of TPM-NH_2 was obtained as light-yellow oil in a 73% yield. ^{1}H NMR (400 MHz, $CDCl_3$), δ (ppm): 7.49-7.47 (d, 6H), 7.28-7.25 (d, 6H), 7.19-7.16 (m, 3H), 2.81-2.78 (t, 2H), 2.22-2.19 (t, 2H). ^{13}C NMR (400 MHz, $CDCl_3$), δ (ppm): 146.1, 128.7, 127.8, 126.2, 70.7, 46.6, 42.8. HRMS: *m*/*z*: calculated for $[C_{21}H_{23}N_2]^+$: 303.1861 $[M+H]^+$; found: 303.1866 $[M+H]^+$.

(E)-2-[(4-chlorobenzylidene)amino]-N-tritylethan-1-amine (TPMI-Cl)

TPM-NH_2 (1.00 g, 3.31 mmol) and 4-chlorobenzaldehyde (0.51 g, 3.64 mmol) with a molar ratio of 1:1.1 were added into a 100 mL two-necked flask with 30 mL ethanol. Several drops of acetic acid were added to the mixture as the catalyst. The mixture was stirred at 70 °C for 12 h under nitrogen gas (Supplementary Scheme 1). The mixture was cooled to room temperature and evaporated under reduced pressure. The product was purified through recrystallization in an ethanol/water mixture at least three times. 0.96 g of TPMI-Cl was obtained as colorless crystals in a 69% yield. ^{1}H NMR (400 MHz, $CDCl_3$), δ (ppm): 8.29 (s, 1H), 7.67-7.65 (d, 2H), 7.48-7.46 (d, 6H), 7.39-7.37 (d, 2H), 7.27-7.23 (m, 6H), 7.19-7.15 (m, 3H), 3.75-3.72 (t, 2H), 2.50-2.47 (d, 2H). ^{13}C NMR (400 MHz, $CDCl_3$), δ (ppm): 160.5, 146.1, 136.6 134.7, 129.3, 128.9, 128.7, 127.8, 126.2, 70.8, 61.9, 44.3. HRMS: *m*/*z*: calculated for $[C_{28}H_{26}N_2Cl]^+$: 425.1785 $[M+H]^+$; found: 425.1792 $[M+H]^+$.

(E)-2-[(4-bromobenzylidene)amino]-N-tritylethan-1-amine (TPMI-Br)

TPM-NH_2 (1.00 g, 3.31 mmol) and 4-bromobenzaldehyde (0.67 g, 3.64 mmol) with a molar ratio of 1:1.1 were added into a 100 mL two-necked flask with 30 mL ethanol. Several drops of acetic acid were added to the mixture as the catalyst. The mixture was stirred at 70 °C for 12 h under nitrogen gas (Supplementary Scheme 1). The mixture was cooled to room temperature and evaporated under reduced pressure. The product was purified through recrystallization in an ethanol/water mixture at least three times. 1.09 g of TPMI-Br was obtained as colorless crystals in a 70% yield. ^{1}H NMR (400 MHz, $CDCl_3$), δ (ppm): 8.27 (s, 1H), 7.60-7.58 (d, 2H), 7.55-7.53 (d, 2H), 7.48-7.46 (d, 6H), 7.27-7.23 (m, 6H), 7.18-7.15 (m, 3H), 3.74-3.71 (t, 2H), 2.49-2.46 (d, 2H). ^{13}C NMR (400 MHz, $CDCl_3$), δ (ppm): 160.6, 146.1, 135.1, 131.8, 129.5, 128.7, 127.8, 126.2, 125.0. HRMS: *m*/*z*: calculated for $[C_{28}H_{26}N_2Br]^+$: 469.1279 $[M+H]^+$; found: 469.1289 $[M+H]^+$.

(E)-2-[(4-iodobenzylidene)amino]-N-tritylethan-1-amine (TPMI-I)

TPM-NH_2 (1.00 g, 3.31 mmol) and 4-iodobenzaldehyde (0.84 g, 3.64 mmol) with a molar ratio of 1:1.1 were added into a 100 mL two-necked flask with 30 mL absolute ethanol. Several drops of acetic acid were added to the mixture as the catalyst. The mixture was stirred at 70 °C for 12 h under nitrogen gas (Supplementary Scheme 1). The mixture was cooled to room temperature and evaporated under reduced pressure. The product was purified through recrystallization in an ethanol/water mixture at least three times. 1.19 g of TPMI-I was obtained as colorless crystals in a 70% yield. ^{1}H NMR (400 MHz, $CDCl_3$), δ (ppm): 8.25 (s, 1H), 7.76-7.74 (d, 2H), 7.48-7.44 (m, 8H), 7.27-7.23 (m, 6H), 7.18-7.15 (m, 3H), 3.74-3.71 (t, 2H), 2.49-2.47 (d, 2H). ^{13}C NMR (400 MHz, $CDCl_3$), δ (ppm): 160.9, 146.1, 137.8, 135.7, 129.6, 128.7, 127.8, 126.2, 97.2, 70.8, 62.0, 44.3. HRMS: *m*/*z*: calculated for $[C_{28}H_{26}N_2I]^+$: 517.1141 $[M+H]^+$; found: 517.1145 $[M+H]^+$.

Recrystallization protocol

The obtained products (TPMI-Cl, TPM-Br, and TPMI-I) were added to a 250 mL two-necked flask with 80 mL absolute ethanol. The mixture was heated to 80 °C in the oil bath, and about 20 mL of deionized water was added to form a clear and saturated solution. Then the temperature was slowly decreased to room temperature within 6 hours, and the flask was left to stand for another 6 hours. Bulk crystals were washed with absolute ethanol three times and dried in a vacuum oven at 40 °C for 6 hours. Each compound was recrystallized at least three times, and about 10% of the product was lost during each recrystallization process.

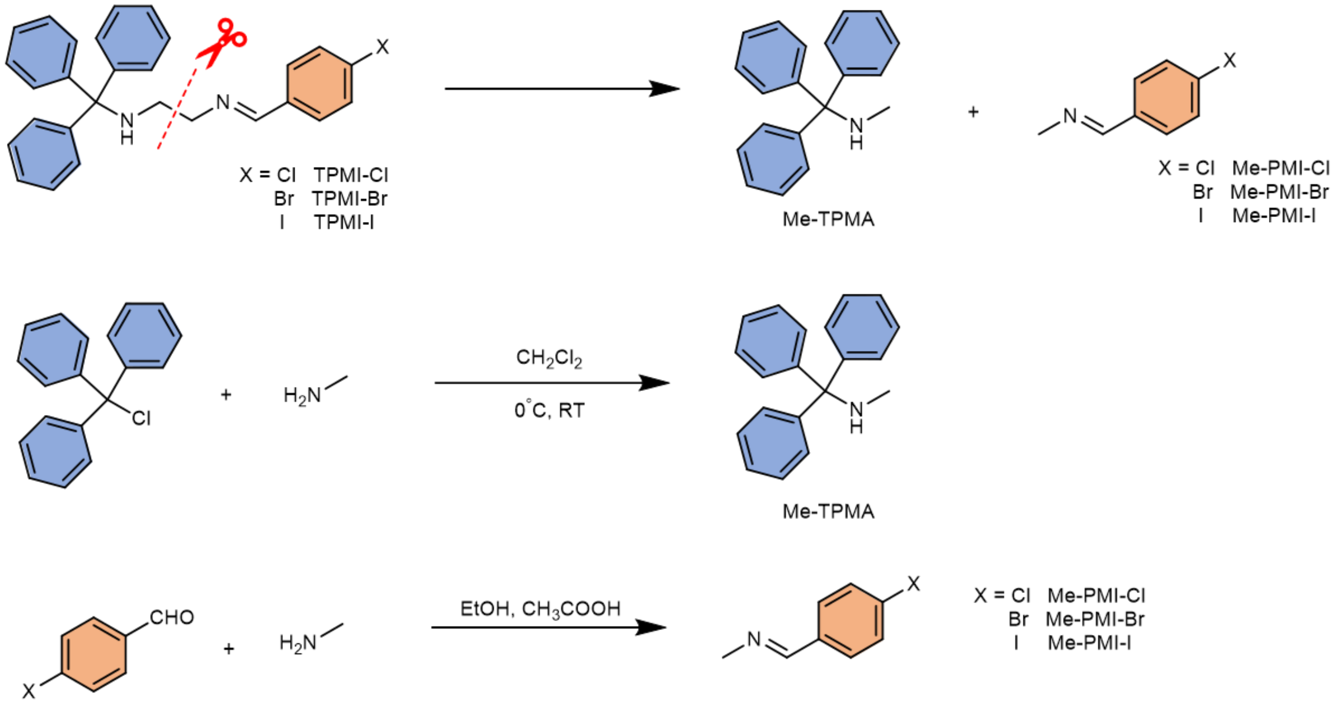

Supplementary Figure 2. Synthetic routes to model compound Me-TPMA and Me-PMI-X (X = Cl, Br, and I).

N-methyl-1,1,1-triphenylmethanamine (Me-TPMA)

3.99 mL aminomethane in absolute ethanol (33 wt. %, 32 mmol, 3.01 g) was added into a 100 mL two-necked flask with 30 mL dichloromethane (DCM) and cooled in ice water to keep the temperature at 0 °C. 1.11 g triphenylmethyl chloride (4 mmol) dissolved in 10 mL DCM was added into the flask drop by drop for 10 mins. The mixture was stirred at 0 °C for 2 h and at room temperature for another 7 h under nitrogen gas (Supplementary Scheme 2). Then the reaction mixture was washed with saturated NaHCO3 solution three times. The organic layer was separated and dried with enough anhydrous sodium sulfate. After filtration, the filtrate was evaporated under reduced pressure, and the crude product was purified on a silica gel column using DCM/hexane mixture (3/5, v/v) as the eluent. 0.59 g of Me-TPMA was obtained as white powder in a 54% yield. ^{1}H NMR (400 MHz, $CDCl_3$), δ (ppm): 7.48-7.46 (d, 6H), 7.29-7.7.25 (t, 6H), 7.20-7.16 (t, 3H), 2.07 (s, 3H). ^{13}C NMR (400 MHz, $CDCl_3$), δ (ppm): 145.8, 128.8, 127.8, 126.2, 71.4, 30.6. HRMS: *m*/*z*: calculated for $[C_{20}H_{20}N]^+$: 275.1596 $[M+H]^+$; found: 274.1598 $[M+H]^+$.

(E)-1-(4-chlorophenyl)-N-methylmethanimine (Me-PMI-Cl)

0.75 mL aminomethane in absolute ethanol (33 wt. %, 6 mmol, 0.56 g) and 4-chlorobenzaldehyde (4 mmol, 0.56 g) with a molar ratio of 1.5:1 were added into a 100 mL two-necked flask with 30 mL ethanol. Several drops of acetic acid were added to the mixture as the catalyst. The mixture was stirred at 70 °C for 12 h under nitrogen gas until the aldehyde was completely reacted (Supplementary Scheme 2). The mixture was cooled to room temperature and evaporated under reduced pressure. The product was washed with absolute ethanol and evaporated under reduced pressure three times. Due to the low boiling points of aminomethane and acetic acid, they were easily removed. 0.38 g of Me-PMI-Cl was obtained as pale-yellow oil in a 58% yield. ^{1}H NMR (400 MHz, $CDCl_3$), δ (ppm): 8.23 (s, 1H), 7.64-7.62 (d, 2H), 7.38-7.36 (d, 2H), 3.51 (s, 3H). ^{13}C NMR (400 MHz,

$CDCl_3$), δ (ppm): 161.1, 136.4, 134.7, 129.1, 128.9, 48.2. HRMS: *m*/*z*: calculated for $[C_8H_9NCl]^+$: 154.0424 $[M+H]^+$; found: 154.0418 $[M+H]^+$.

(E)-1-(4-bromophenyl)-N-methylmethanimine (Me-PMI-Br)

0.75 mL aminomethane in absolute ethanol (33 wt. %, 6 mmol, 0.56 g) and 4-bromobenzaldehyde (4 mmol, 0.74 g) with a molar ratio of 1.5:1 were added into a 100 mL two-necked flask with 30 mL ethanol. Several drops of acetic acid were added to the mixture as the catalyst. The mixture was stirred at 70 °C for 12 h under nitrogen gas until the aldehyde was completely reacted (Supplementary Scheme 2). The mixture was cooled to room temperature and evaporated under reduced pressure. The product was washed with absolute ethanol and evaporated under reduced pressure three times. Due to the low boiling points of aminomethane and acetic acid, they were easily removed. 0.47 g of Me-PMI-Br was obtained as pale-yellow oil in a 60% yield. ^1H NMR (400 MHz, $CDCl_3$), δ (ppm): 8.21 (s, 1H), 7.58-7.52 (dd, 4H), 3.50 (s, 3H). ^{13}C NMR (400 MHz, $CDCl_3$), δ (ppm): 161.2, 135.1, 131.8, 129.3, 124.9, 48.2. HRMS: *m*/*z*: calculated for $[C_8H_9NBr]^+$: 197.9918 $[M+H]^+$; found: 197.9914 $[M+H]^+$.

(E)-1-(4-iodophenyl)-N-methylmethanimine (Me-PMI-I)

0.75 mL aminomethane in absolute ethanol (33 wt. %, 6 mmol, 0.56 g) and 4-iodobenzaldehyde (4 mmol, 0.93 g) with a molar ratio of 1.5:1 were added into a 100 mL two-necked flask with 30 mL ethanol. Several drops of acetic acid were added to the mixture as the catalyst. The mixture was stirred at 70 °C for 12 h under nitrogen gas until the aldehyde was completely reacted (Supplementary Scheme 2). The mixture was cooled to room temperature and evaporated under reduced pressure. The product was washed with absolute ethanol and evaporated under reduced pressure three times. Due to the low boiling points of aminomethane and acetic acid, they were easily removed. 0.50 g of Me-PMI-I was obtained as pale-yellow solid in a 51% yield. ^1H NMR (400 MHz, $CDCl_3$), δ (ppm): 8.20 (s, 1H), 7.76-7.74(d, 2H), 7.43-7.41 (d, 2H), 3.50 (s, 3H). ^{13}C NMR (400 MHz, $CDCl_3$), δ (ppm): 161.4, 137.8, 135.7, 129.4, 97.1, 48.3. HRMS: *m*/*z*: calculated for $[C_8H_8NI]^+$: 244.9701 $[M]^+$; found: 244.9705 $[M]^+$.

Nuclear Magnetic Resonance Spectra

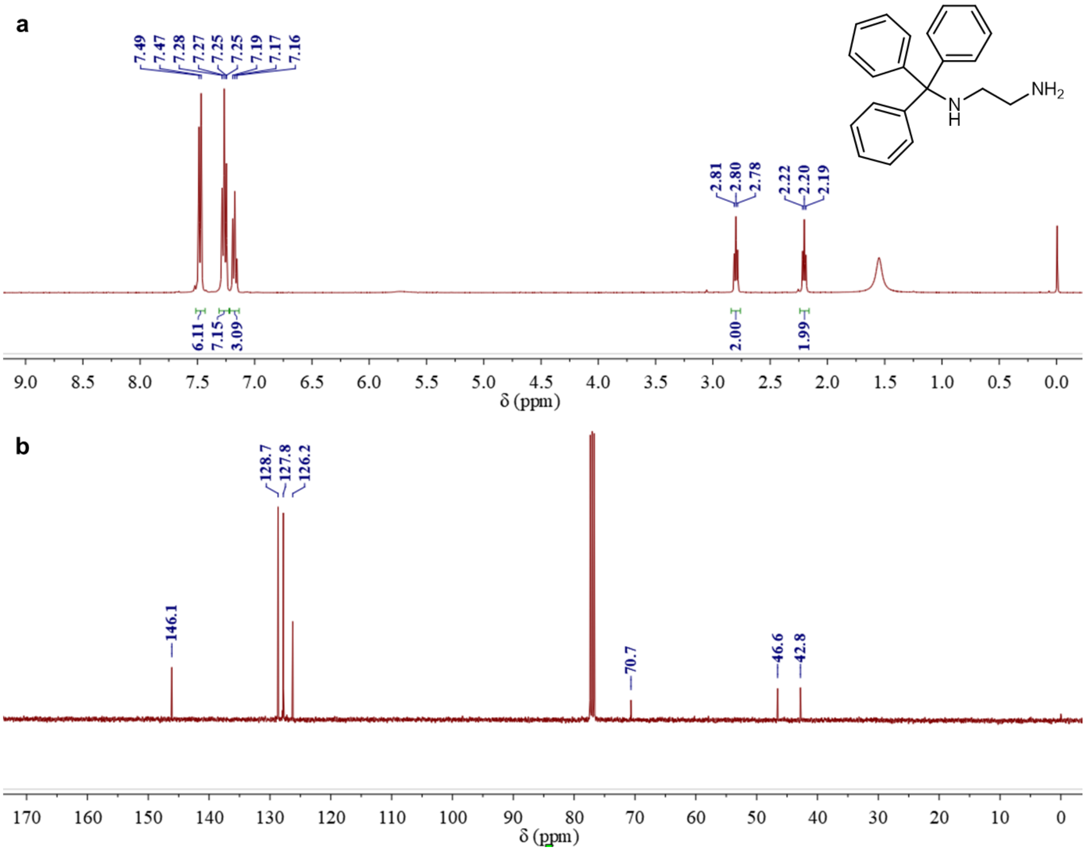

Supplementary Figure 3. a ^1H and **b** ^{13}C NMR spectra of TPM-NH_2 in $CDCl_3$.

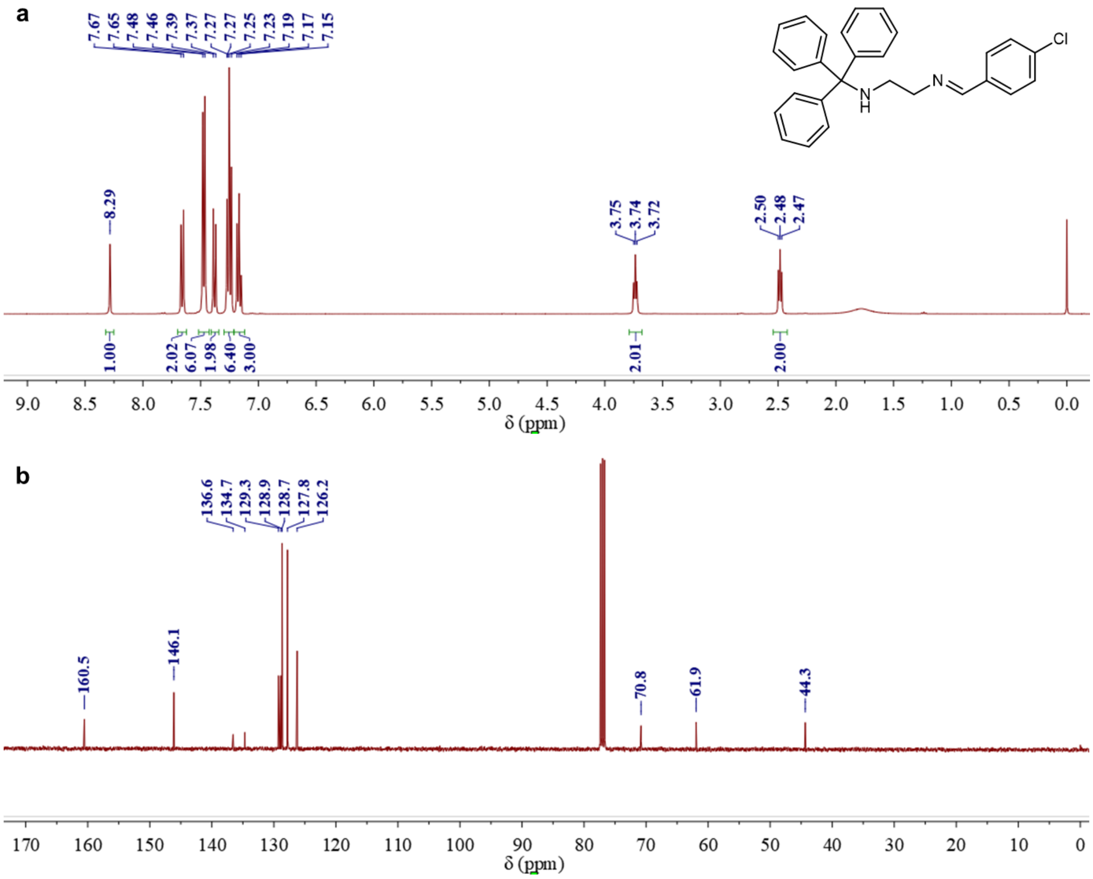

Supplementary Figure 4. a ^{1}H and **b** ^{13}C NMR spectra of TPMI-Cl in $CDCl_3$.

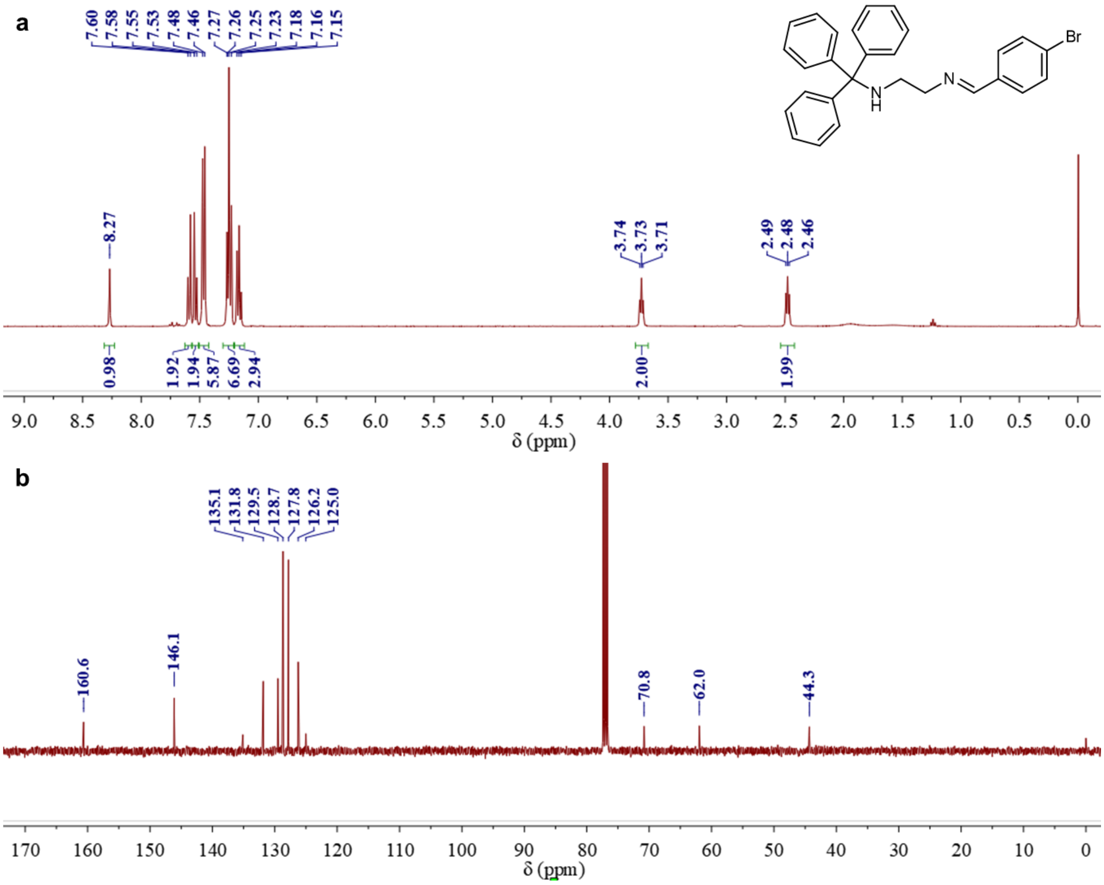

Supplementary Figure 5. a ^{1}H and **b** ^{13}C NMR spectra of TPMI-Br in $CDCl_3$.

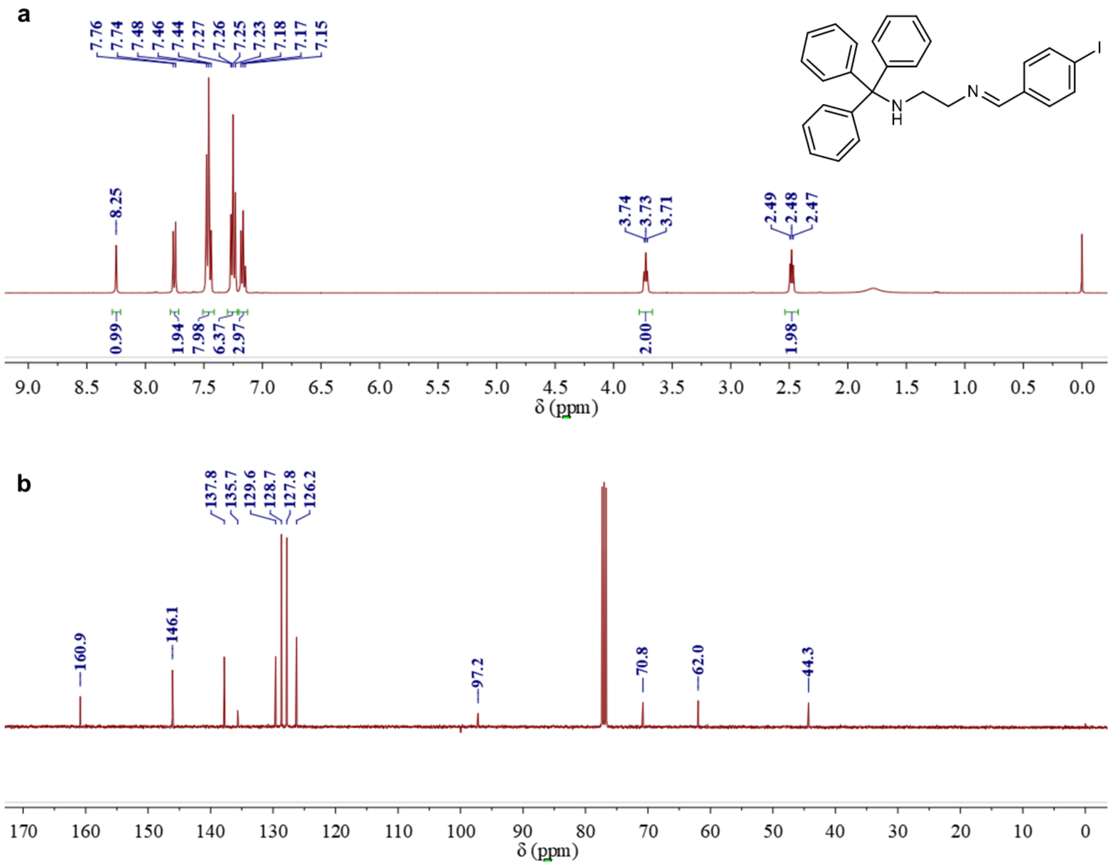

Supplementary Figure 6. a ¹H and **b** ¹³C NMR spectra of TPMI-I in $CDCl_3$.

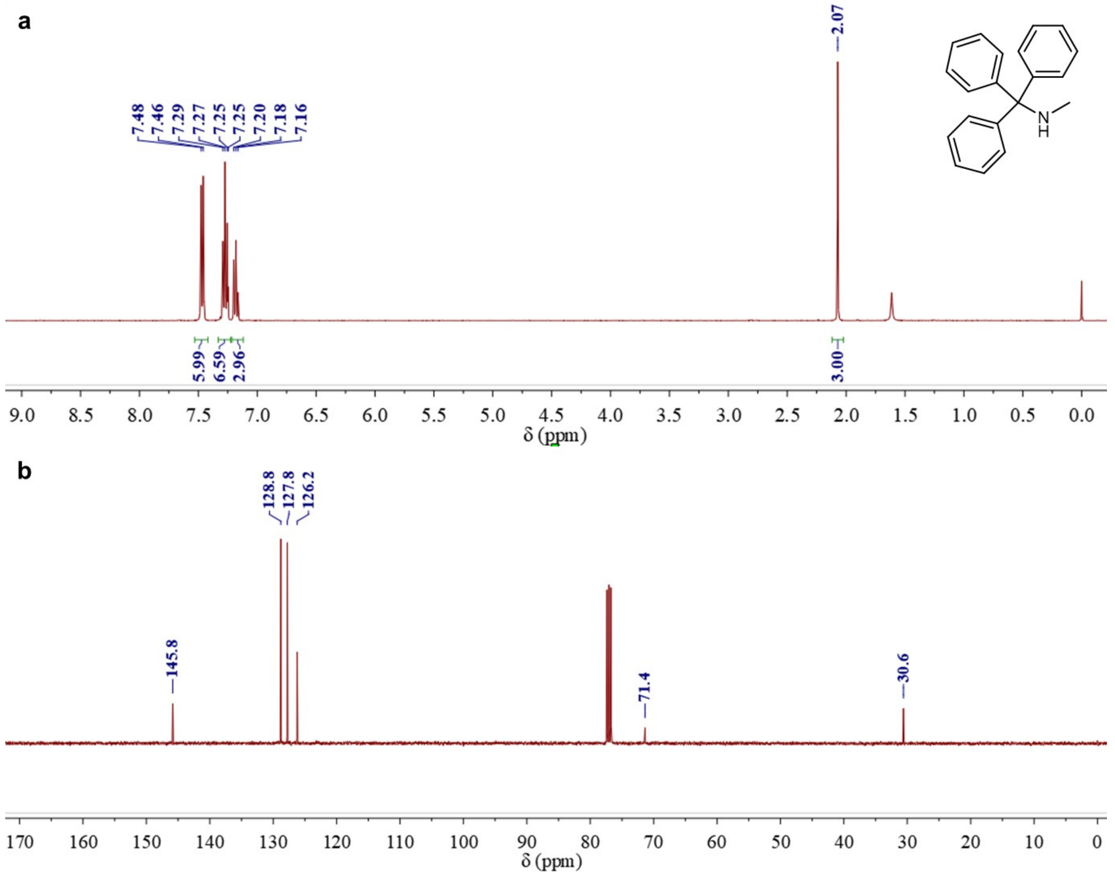

Supplementary Figure 7. a ¹H and **b** ¹³C NMR spectra of Me-TPMA in $CDCl_3$.

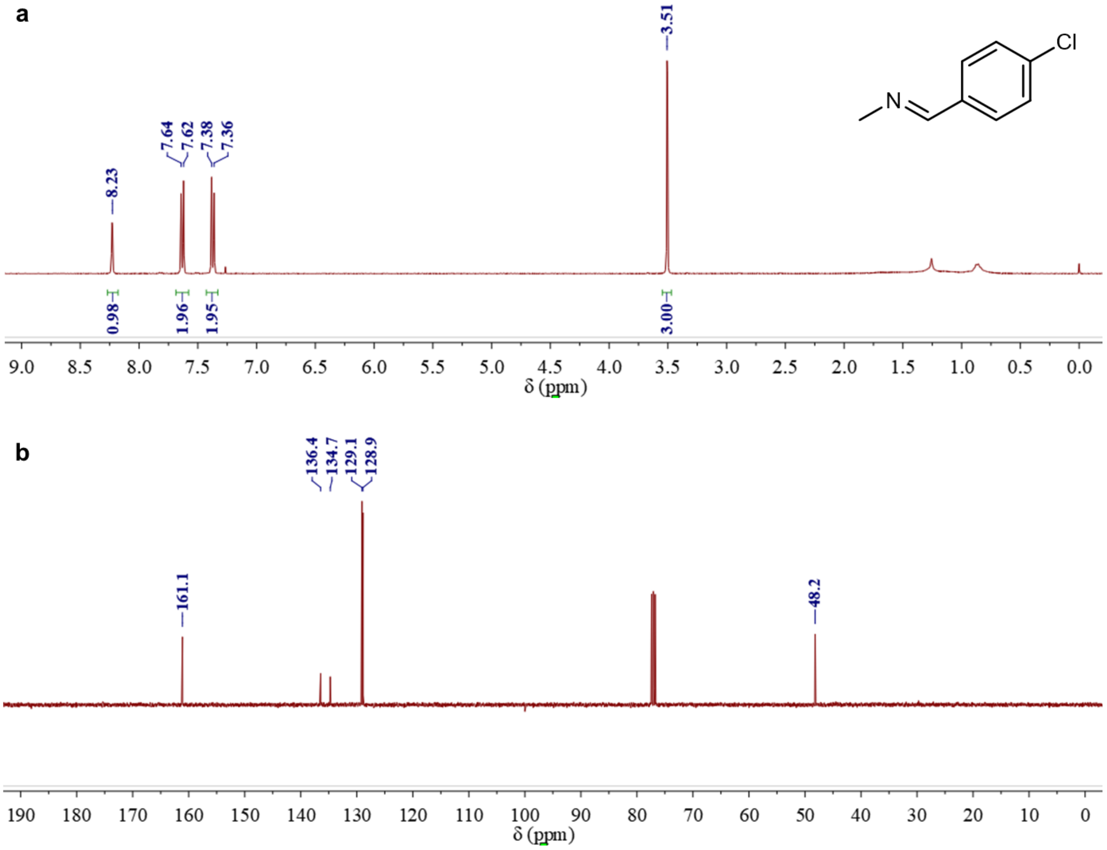

Supplementary Figure 8. a ^{1}H and **b** ^{13}C NMR spectra of Me-PMI-Cl in $CDCl_3$.

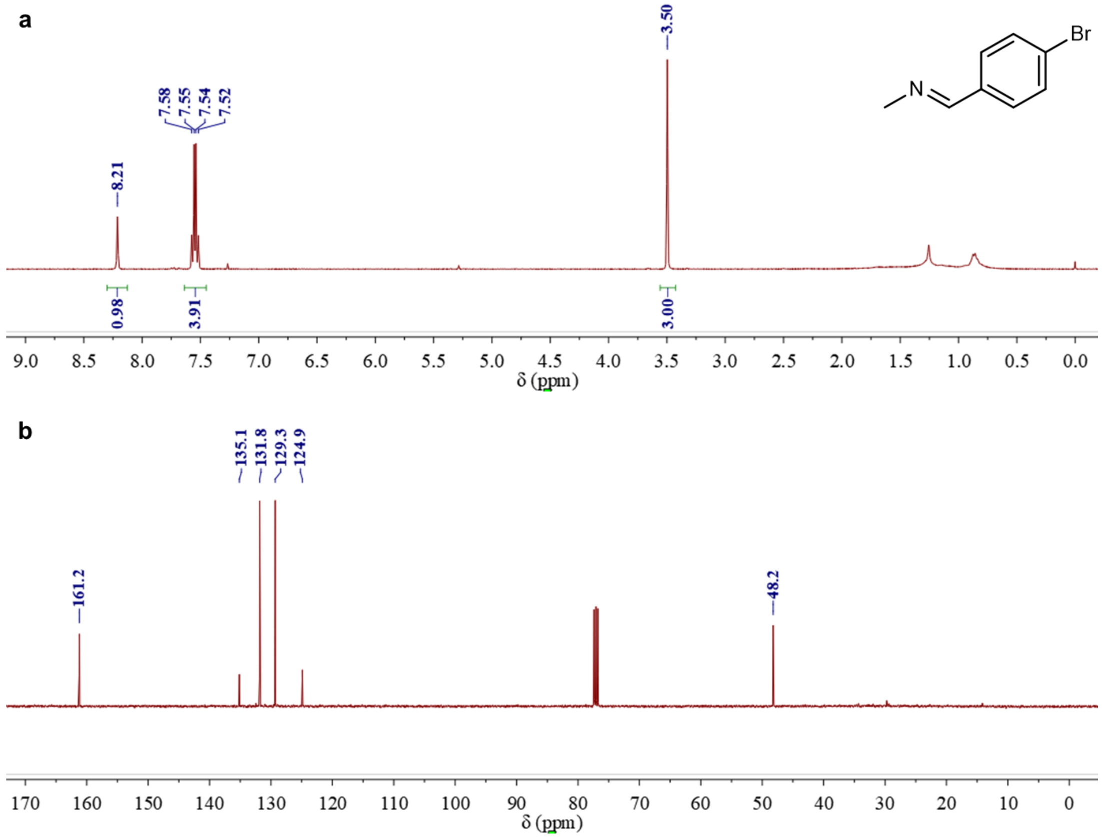

Supplementary Figure 9. a ^{1}H and **b** ^{13}C NMR spectra of Me-PMI-Br in $CDCl_3$.

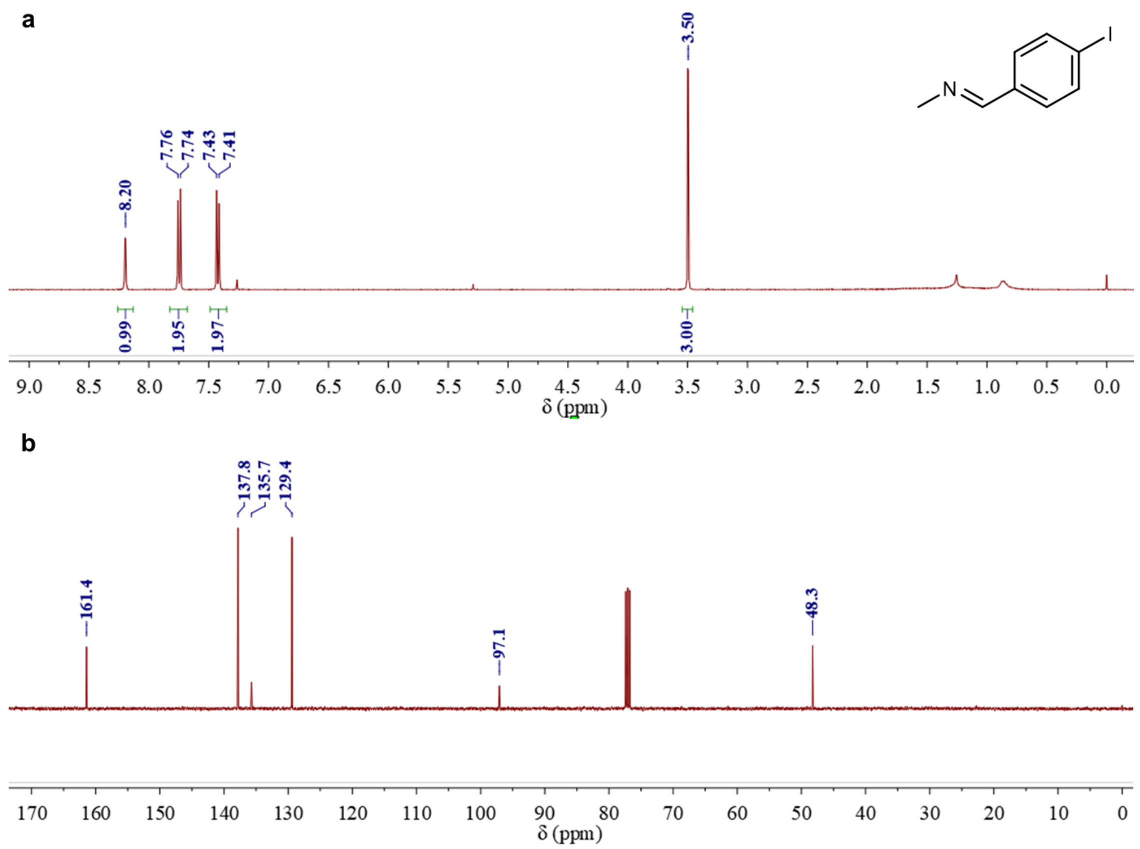

Supplementary Figure 10. a ^{1}H and **b** ^{13}C NMR spectra of Me-PMI-I in $CDCl_3$.

High-Resolution Mass Spectrometry

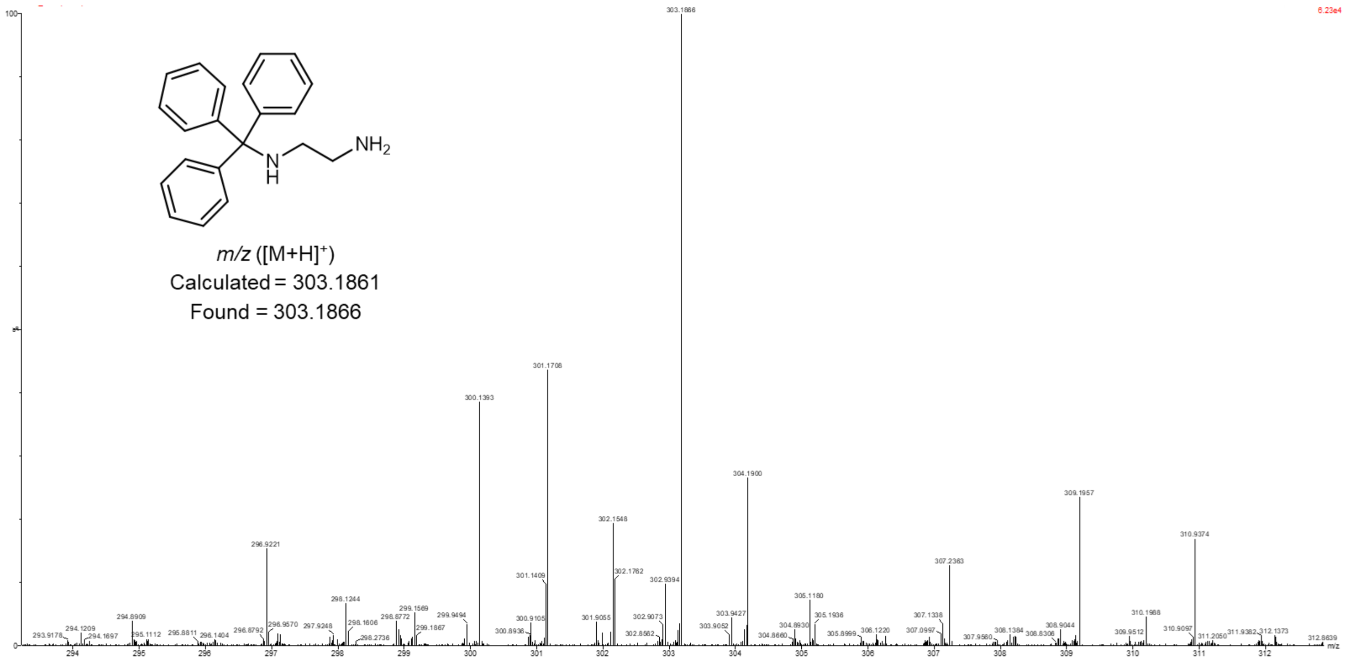

Supplementary Figure 11. High-resolution mass spectrum of TPM-NH_2.

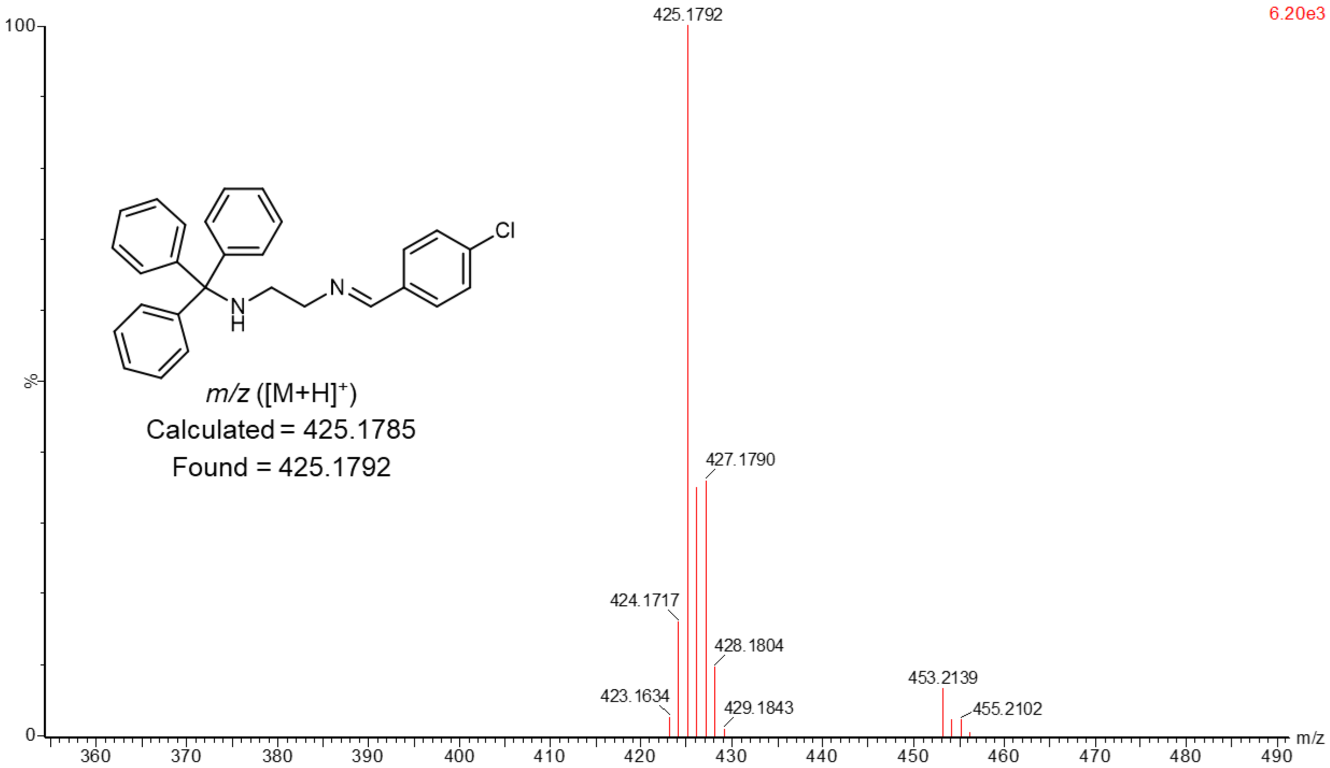

Supplementary Figure 12. High-resolution mass spectrum of TPMI-Cl.

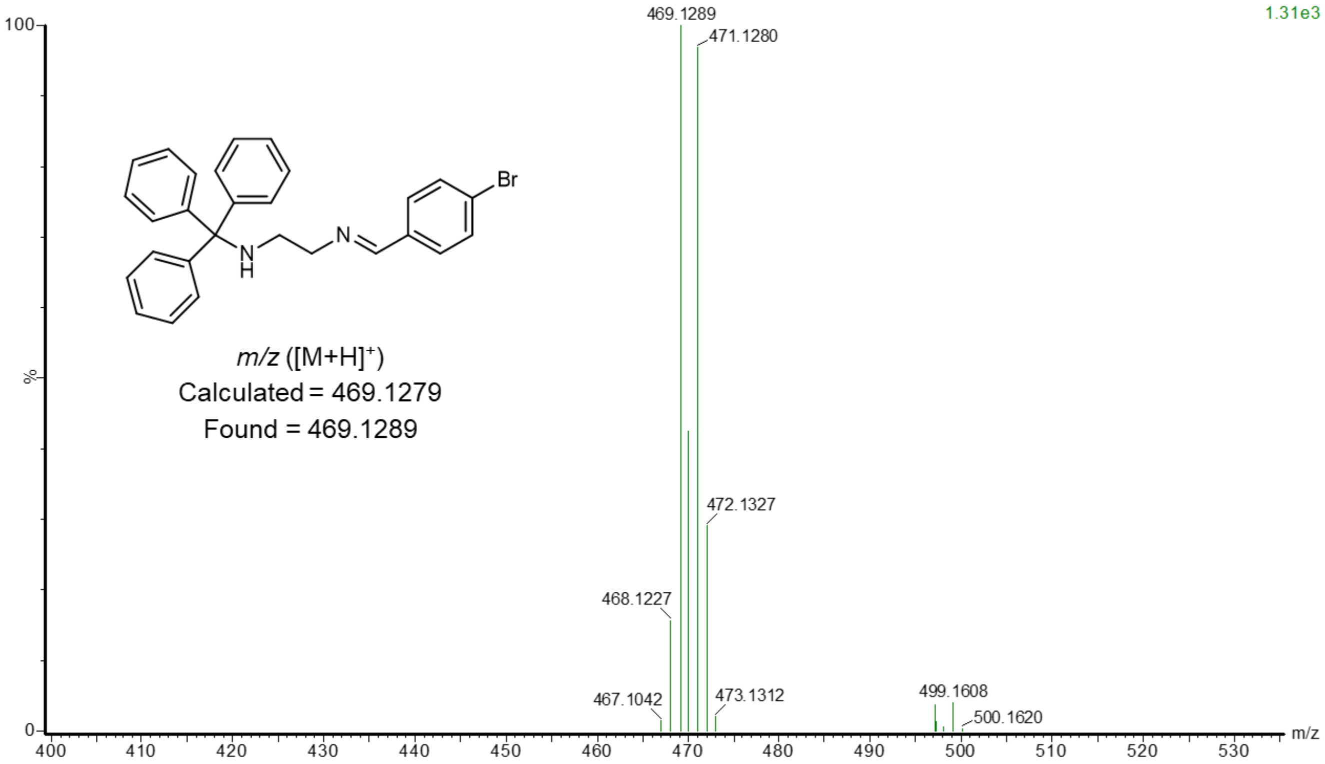

Supplementary Figure 13. High-resolution mass spectrum of TPMI-Br.

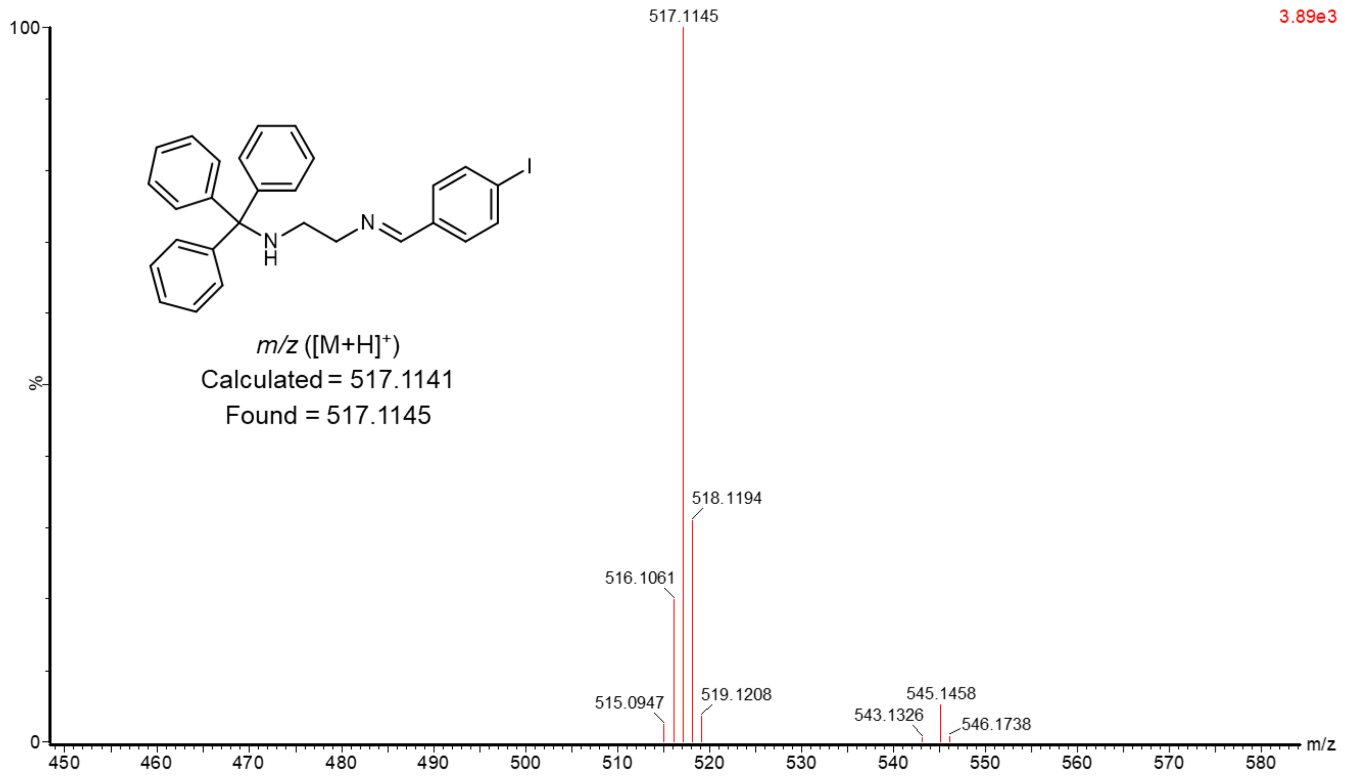

Supplementary Figure 14. High-resolution mass spectrum of TPMI-I.

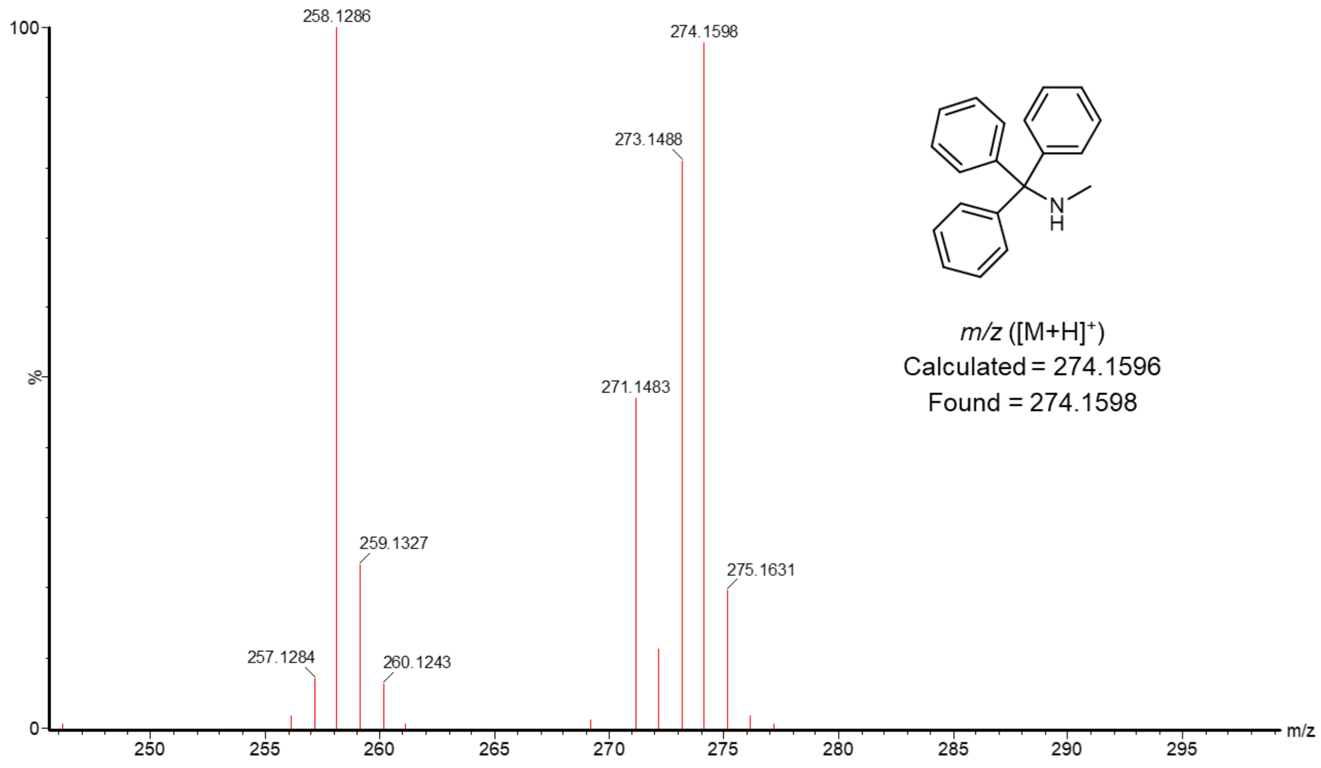

Supplementary Figure 15. High-resolution mass spectrum of Me-TPMA.

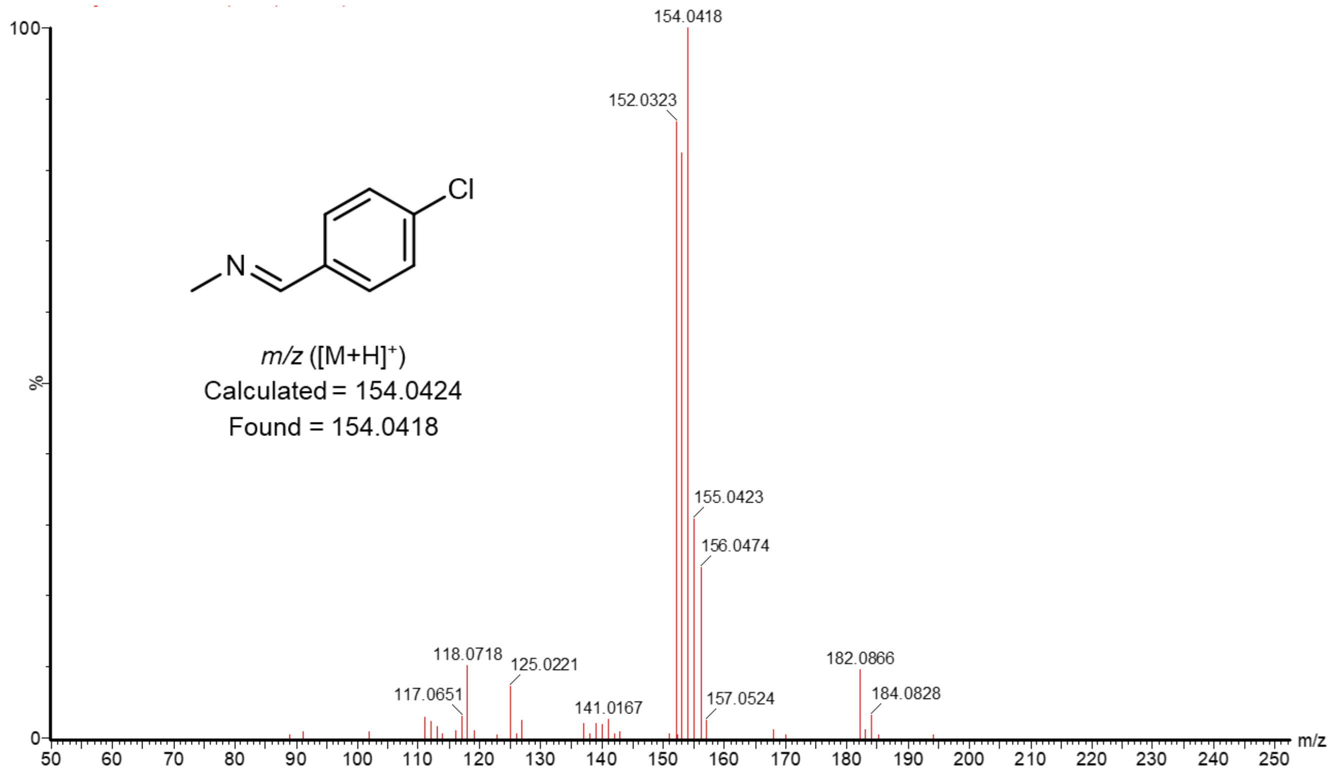

Supplementary Figure 16. High-resolution mass spectrum of Me-PMI-Cl.

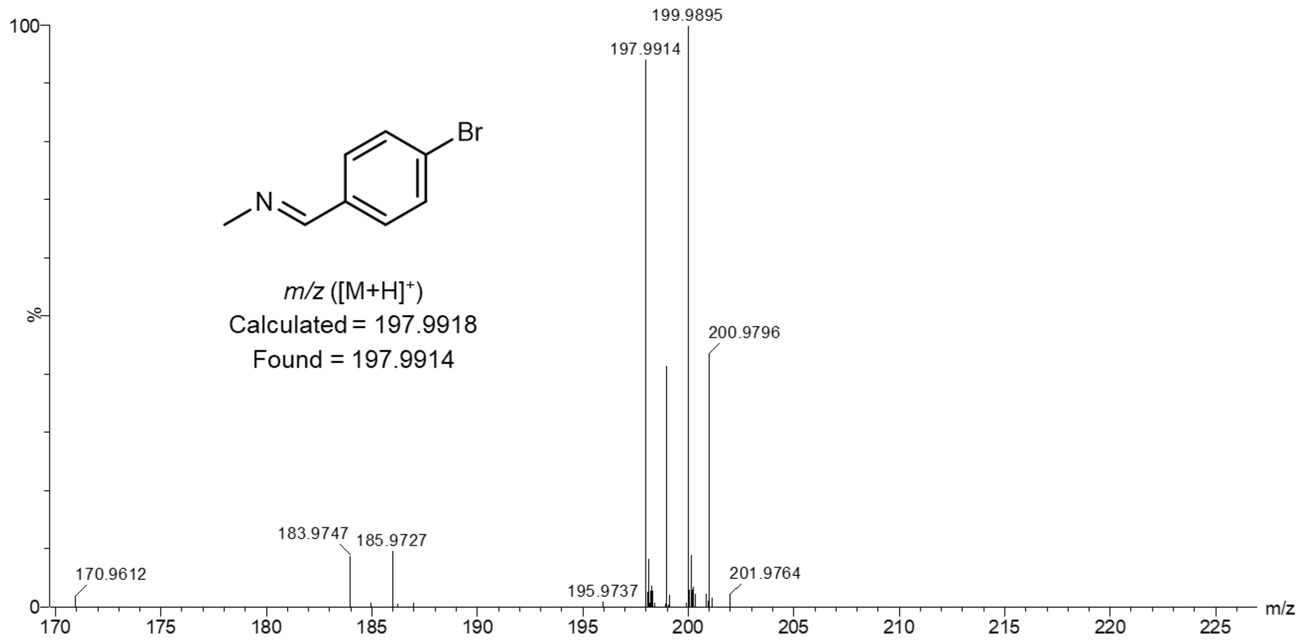

Supplementary Figure 17. High-resolution mass spectrum of Me-PMI-Br.

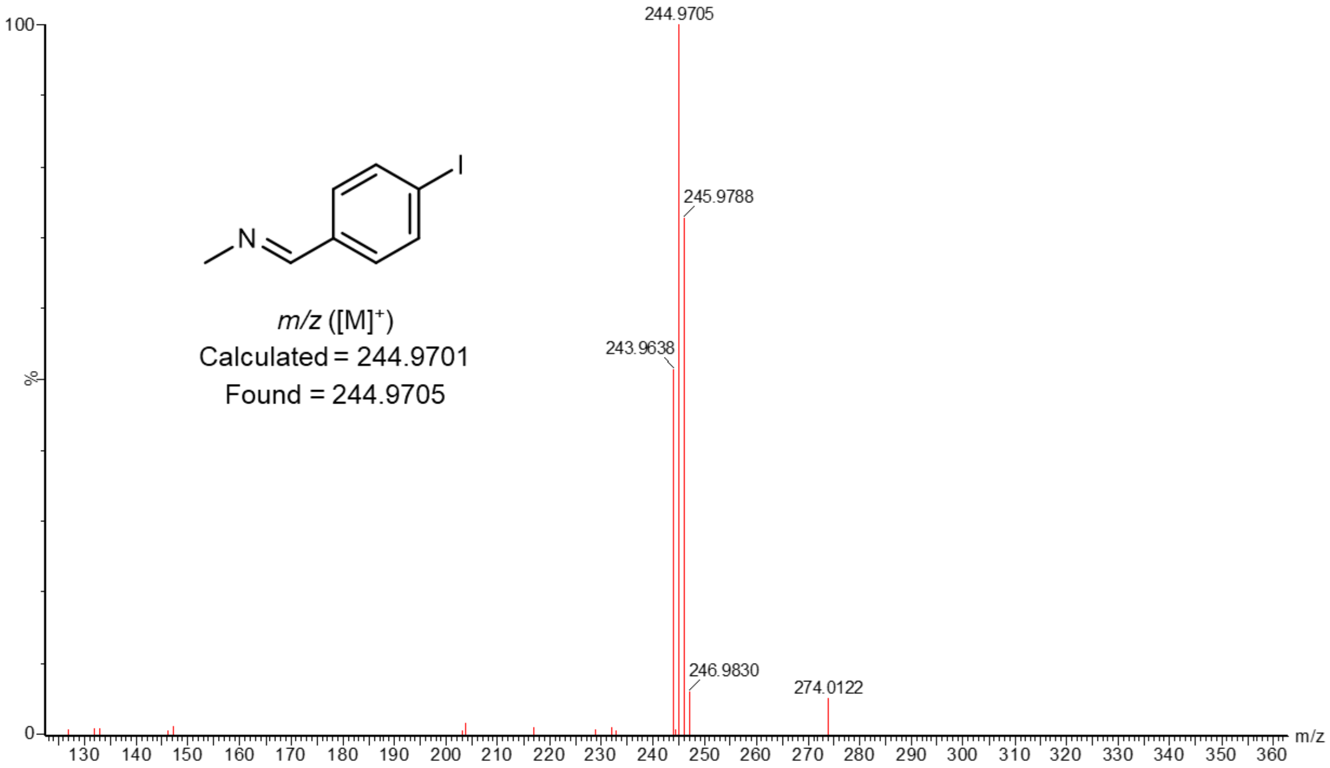

Supplementary Figure 18. High-resolution mass spectrum of Me-PMI-I.

High-Performance Liquid Chromatography

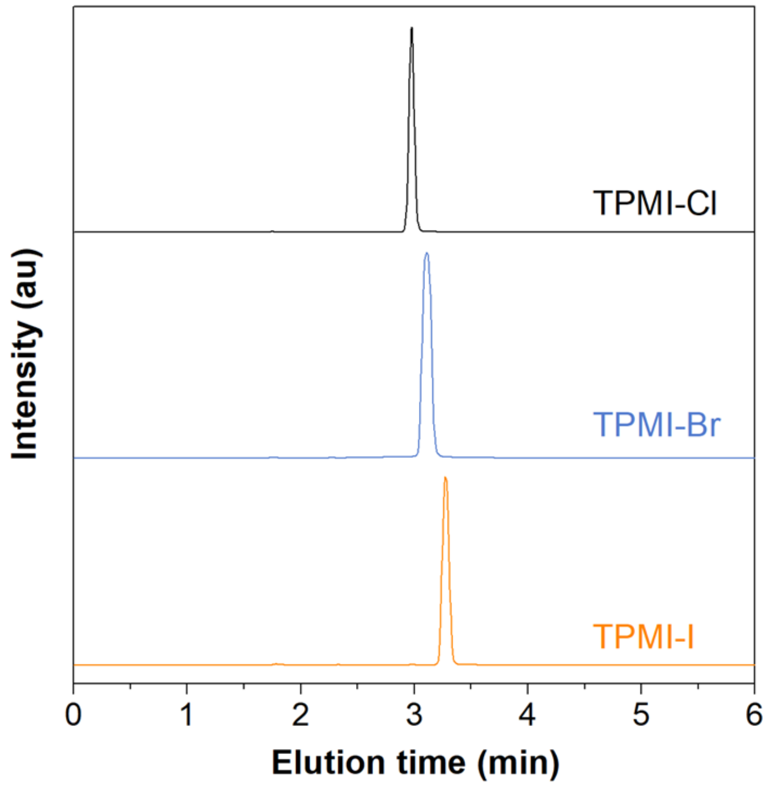

Supplementary Figure 19. High-performance liquid chromatogram of the prepared samples in acetonitrile/water mixtures (9:1, *v*/*v*) with a flow rate of 1.0 mL/min.

Other Characterizations

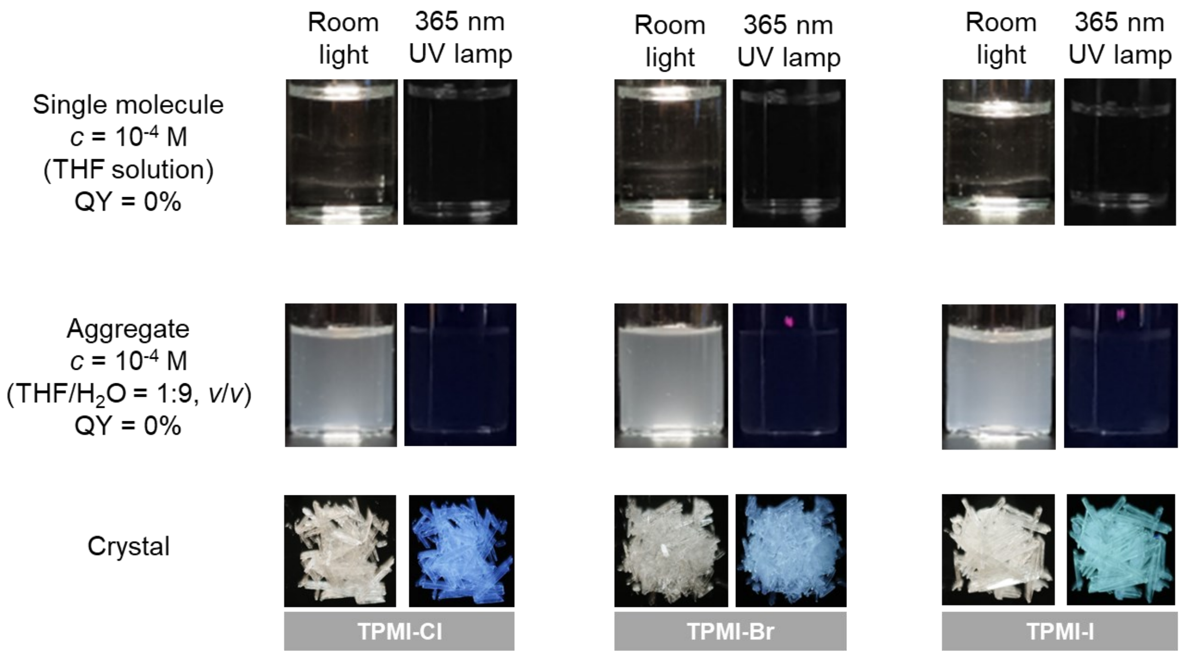

Supplementary Figure 20. Photos of single molecules (in THF solution), aggregates (in a THF/H_2O mixture), and bulk crystals of TPMI-Cl, TPMI-Br, and TPMI-I taken under room light and irradiation of a 365 nm UV lamp.

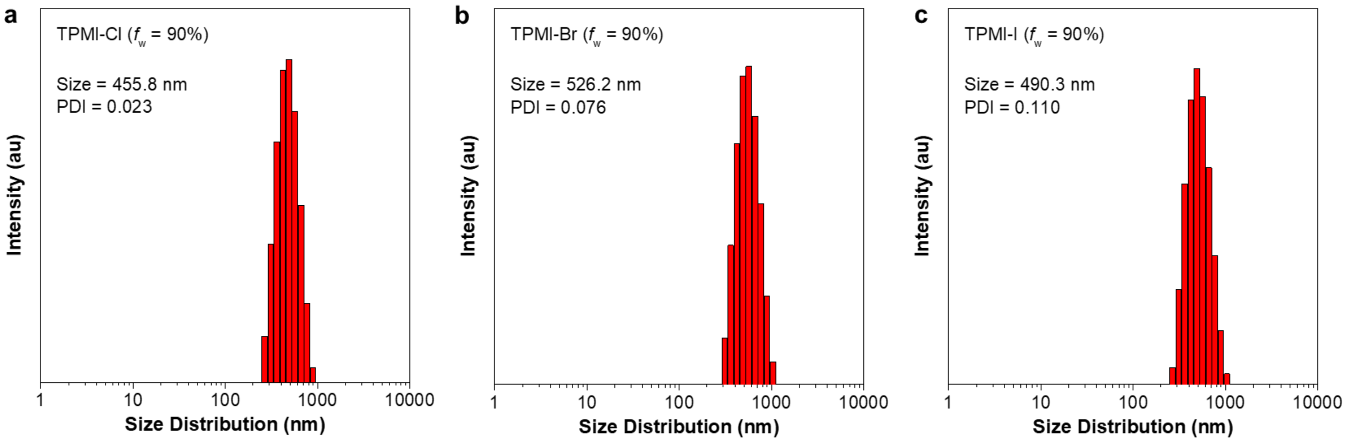

Supplementary Figure 21. The size distribution of **a** TPMI-Cl, **b** TPMI-Br, and **c** TPMI-I in THF/water mixtures with a water fraction of 90%. Concentration = 10^{-4} M. PDI = polydispersity index.

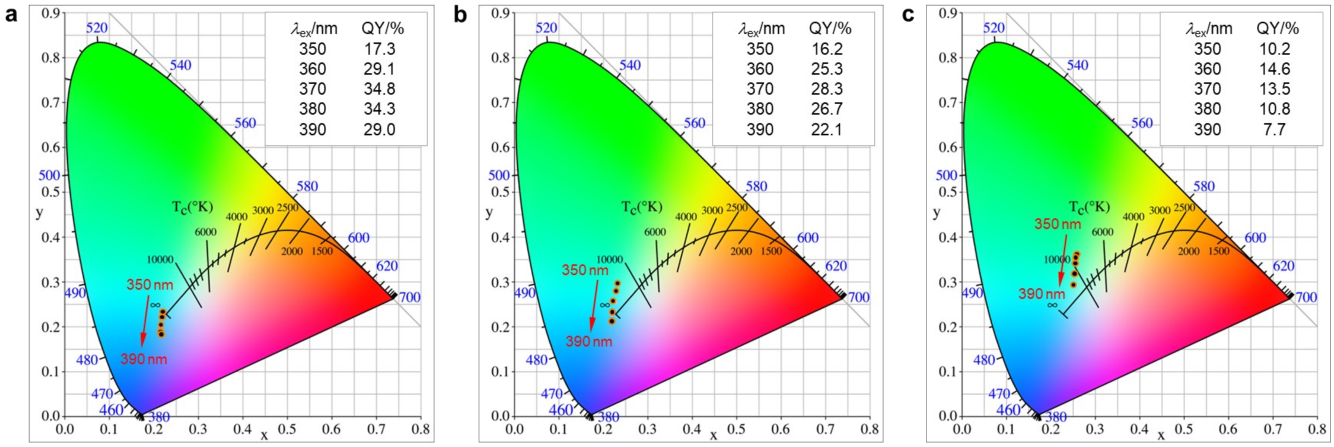

Supplementary Figure 22. The Commission Internationale de L'Eclairage chromaticity coordinate and absolute quantum yield (QY) of **a** TPMI-Cl, **b** TPMI-Br, and **c** TPMI-I in the crystalline state at different excitation wavelengths (λ_{ex}).

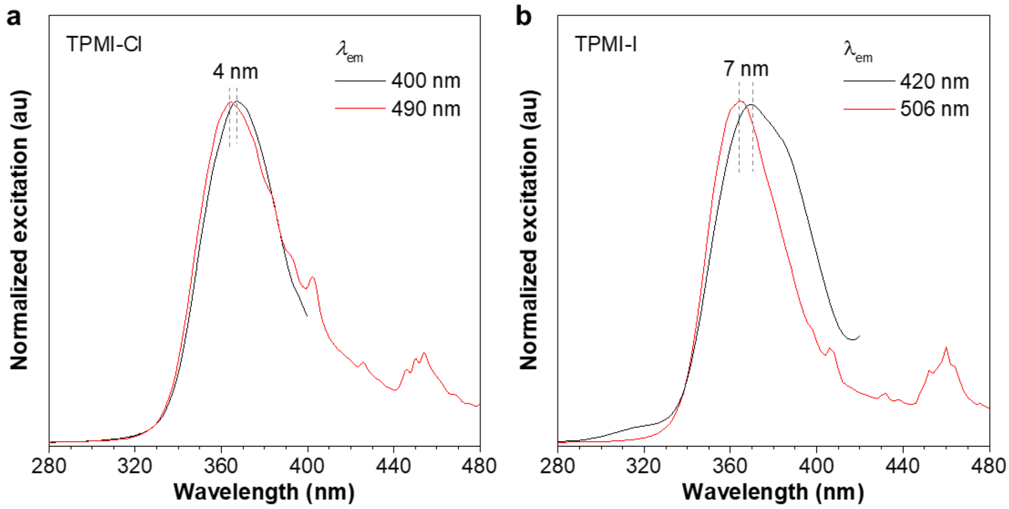

Supplementary Figure 23. Excitation spectra of **a** TPMI-Cl taken at 400 nm and 490 nm, and **b** TPMI-I taken at 420 nm and 506 nm, respectively.

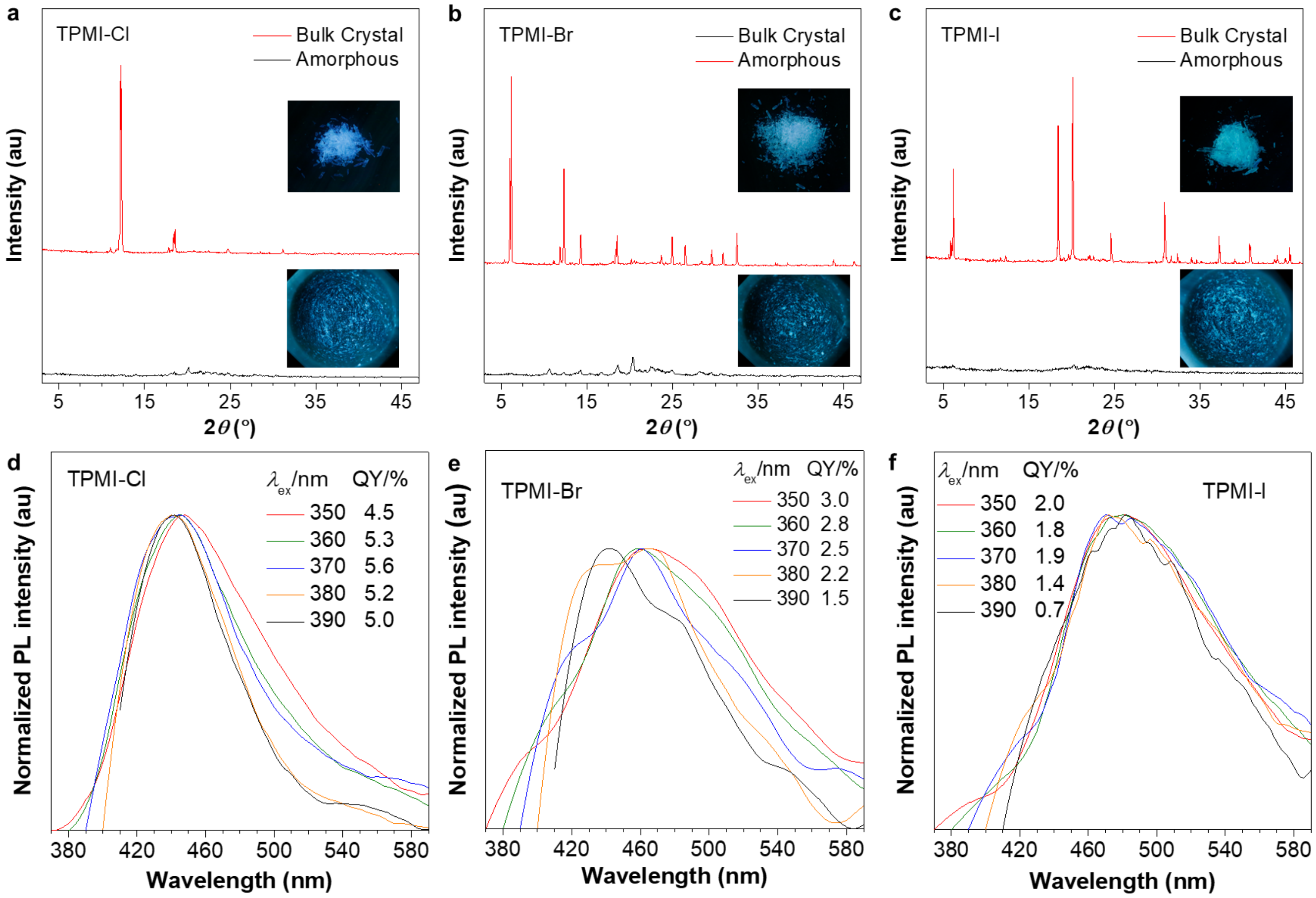

Supplementary Figure 24. a–c Powder X-ray diffraction patterns of (**a**) TPMI-Cl, (**b**) TPMI-Br, and (**c**) TPMI-I before and after grinding. Inset: Photos of the compounds before and after grinding taken under the 365 nm UV irradiation. **d–f** Normalized photoluminescence spectra and quantum yields (QY) of (**d**) TPMI-Cl, (**e**) TPMI-Br, and (**f**) TPMI-I in the amorphous state at different excitation wavelengths (λ_{ex}).

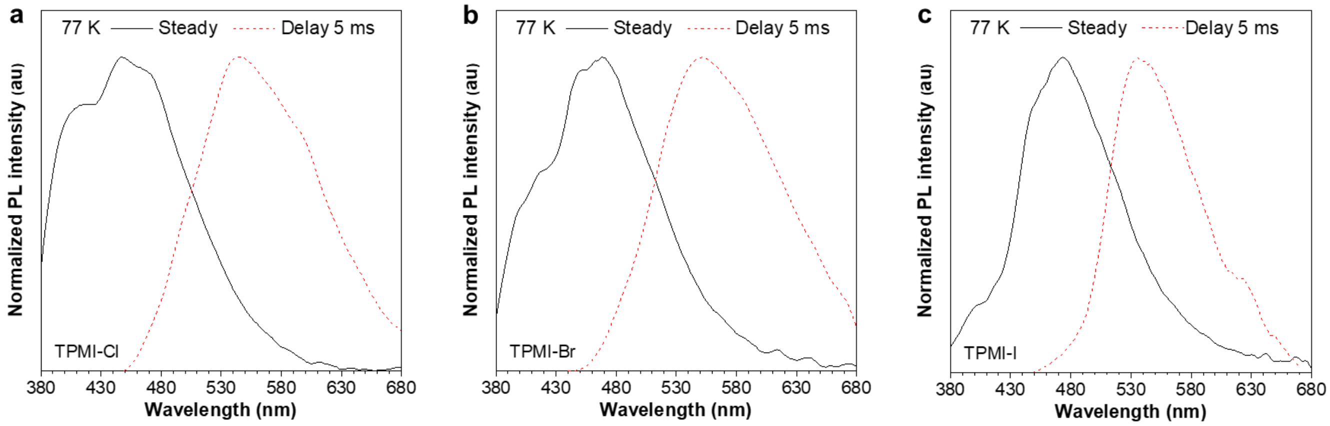

Supplementary Figure 25. Normalized steady and delayed photoluminescence spectra of **a** TPMI-Cl, **b** TPMI-Br, and **c** TPMI-I in the crystalline state at 77 K. λ_{ex} = 365 nm.

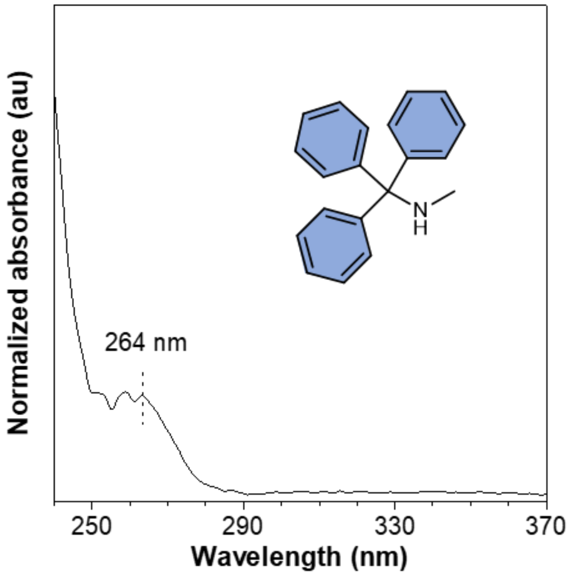

Supplementary Figure 26. Normalized absorption spectra of *N*-methyl-triphenylmethanamine (Me-TPMA) in THF solution.

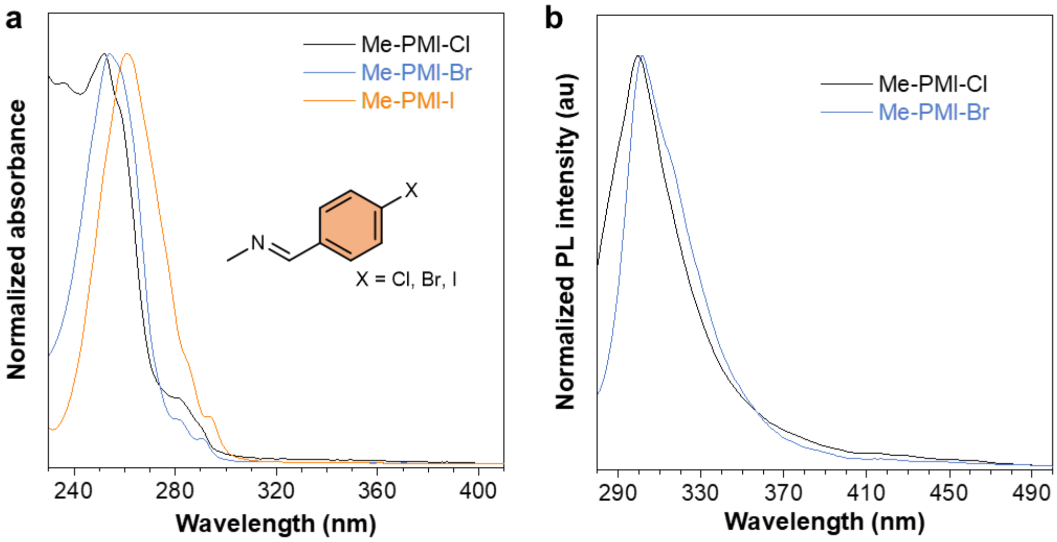

Supplementary Figure 27. a Normalized absorption spectra of halogen-substituted *N*-methyl-phenylmethanimine (Me-PMI-X, X = Cl, Br, and I) as model compounds in THF solution. **b** Normalized photoluminescence spectra of the model compounds in THF solution at room temperature. Me-PMI-I was non-emissive due to the heavy-atom effect of iodine.

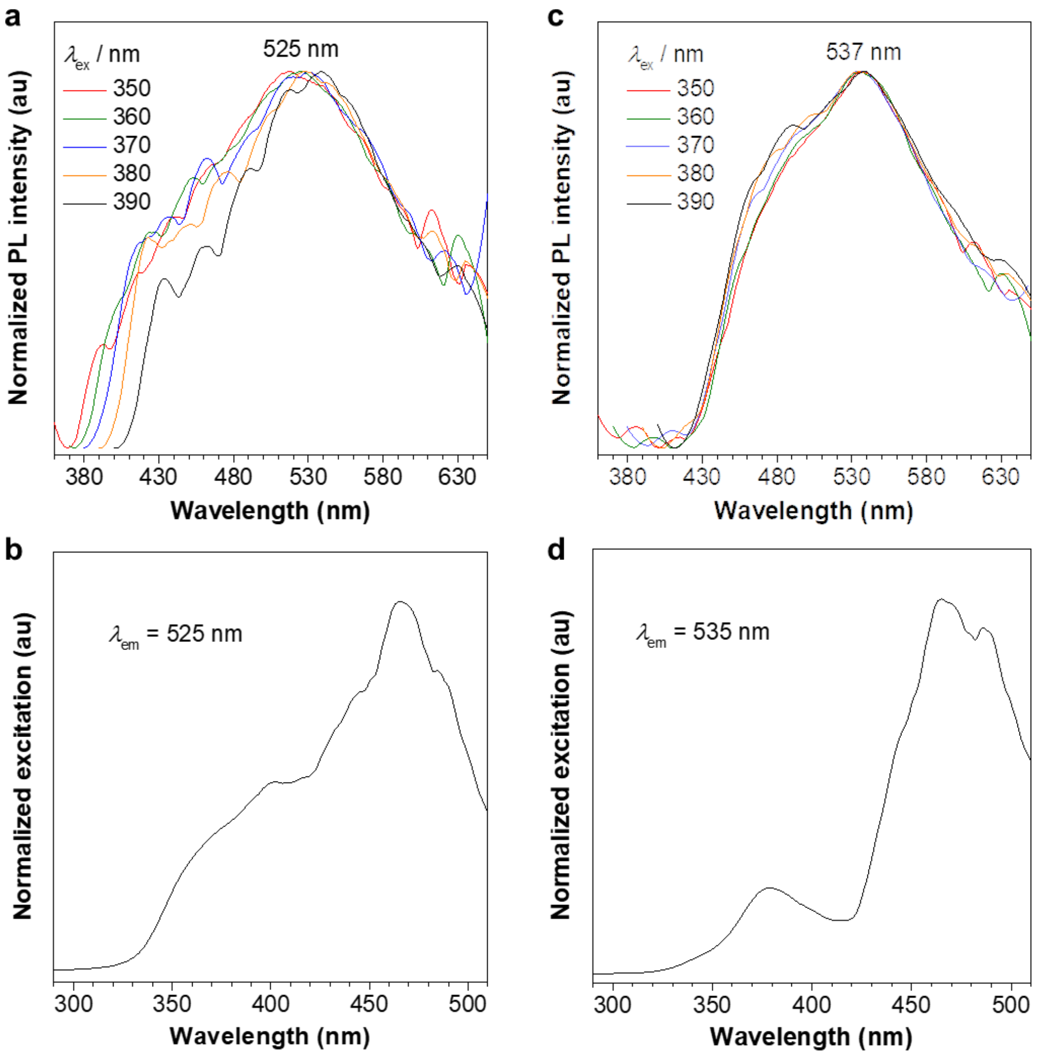

Supplementary Figure 28. a, c Normalized photoluminescence spectra of the (**a**) Me-TPMA/Me-PMI-Cl and (**c**) Me-TPMA/Me-PMI-I mixture with a molar ratio of 1:1 in the solid state at different excitation wavelengths (λ_{ex}). **b, d** Excitation spectra of the (**b**) Me-TPMA/Me-PMI-Cl and (**d**) Me-TPMA/Me-PMI-I mixture in the solid state at their corresponding emission maximums (λ_{em}).

Supplementary Table 1. Summary of crystallographic data and structural refinement of the synthesized compounds TPMI-X (X = Cl, Br, and I).

Compound	TPMI-Cl	TPMI-Br	TPMI-I
CCDC number	2122661	2122660	2122662
Empirical formula	$C_{28}H_{25}ClN_2$	$C_{28}H_{25}BrN_2$	$C_{28}H_{25}IN_2$
Formula weight	424.95	469.41	516.40
Temperature/K	100.01(10)	100.01(10)	100.01(10)
Crystal system	orthorhombic	orthorhombic	monoclinic
Space group	$Pna2_1$	$Pna2_1$	$P2_1$
a/Å	9.23948(19)	9.21690(10)	9.24223(13)
b/Å	28.3116(6)	28.6942(5)	8.67304(12)
c/Å	8.65925(19)	8.64500(10)	14.4498(2)
α/°	90	90	90
β/°	90	90	93.7673(13)
γ/°	90	90	90
Volume/Å^3	2265.12(8)	2286.36(5)	1155.77(3)
Z	4	4	2
Density/ g·cm^{-3}	1.246	1.364	1.484
F(000)	896.0	968.0	520.0
μ/mm^{-1}	1.610	2.568	11.002
Goodness-of-fit on F^2	1.049	1.062	1.029
R_1 [I>=2σ (I)][a]	0.0313	0.0292	0.0261
wR_2 [I>=2σ (I)][b]	0.0813	0.0671	0.0581
R_1 [all data][a]	0.0317	0.0323	0.0282
wR_2 [all data][b]	0.0816	0.0685	0.0593

[a]$R_1 = F_o - F_c/F_o$

[b]$wR_2 = \{w[(F_o)^2 - (F_c)^2]^2/w[(F_o)^2]^2\}^{1/2}$

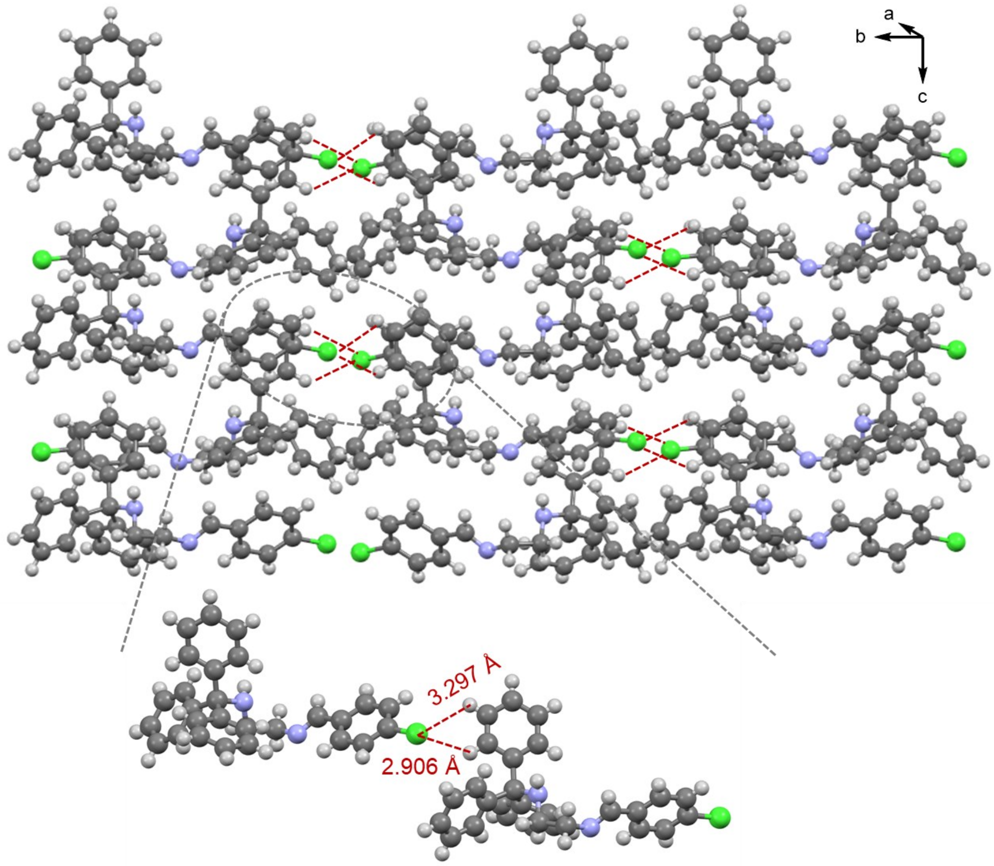

Supplementary Figure 29. Crystal packing diagram of TPMI-Cl.

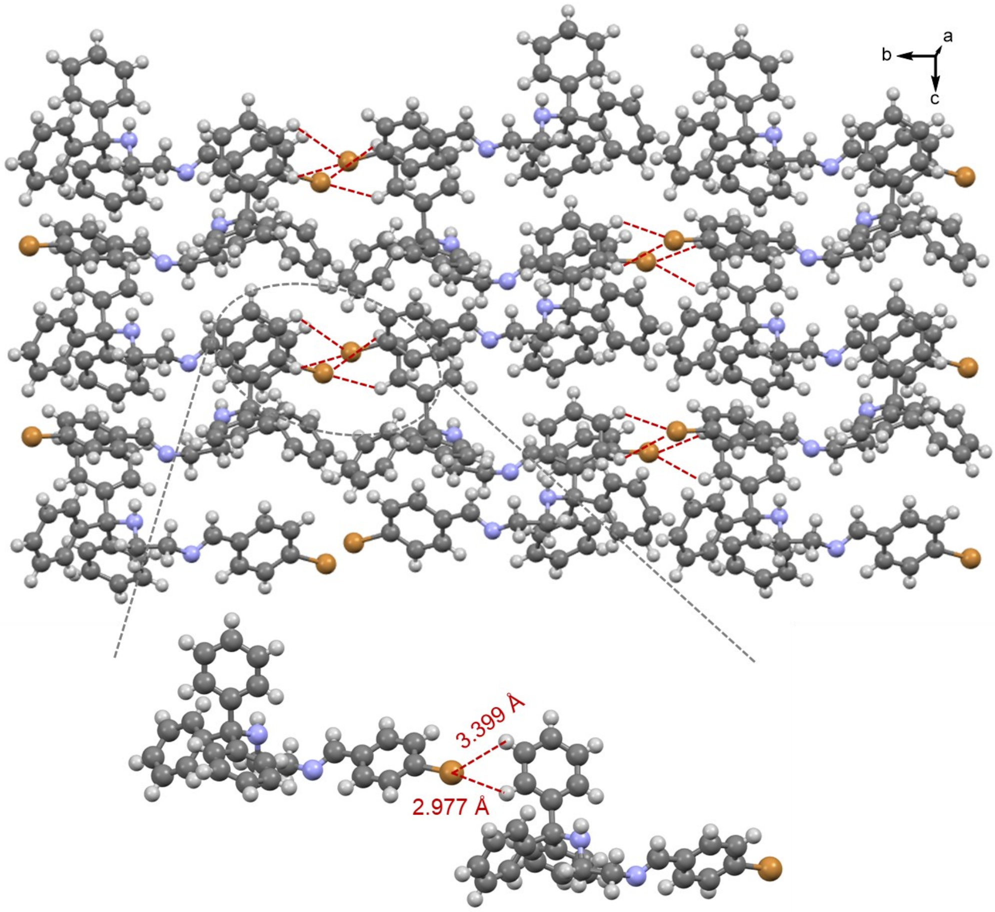

Supplementary Figure 30. Crystal packing diagram of TPMI-Br.

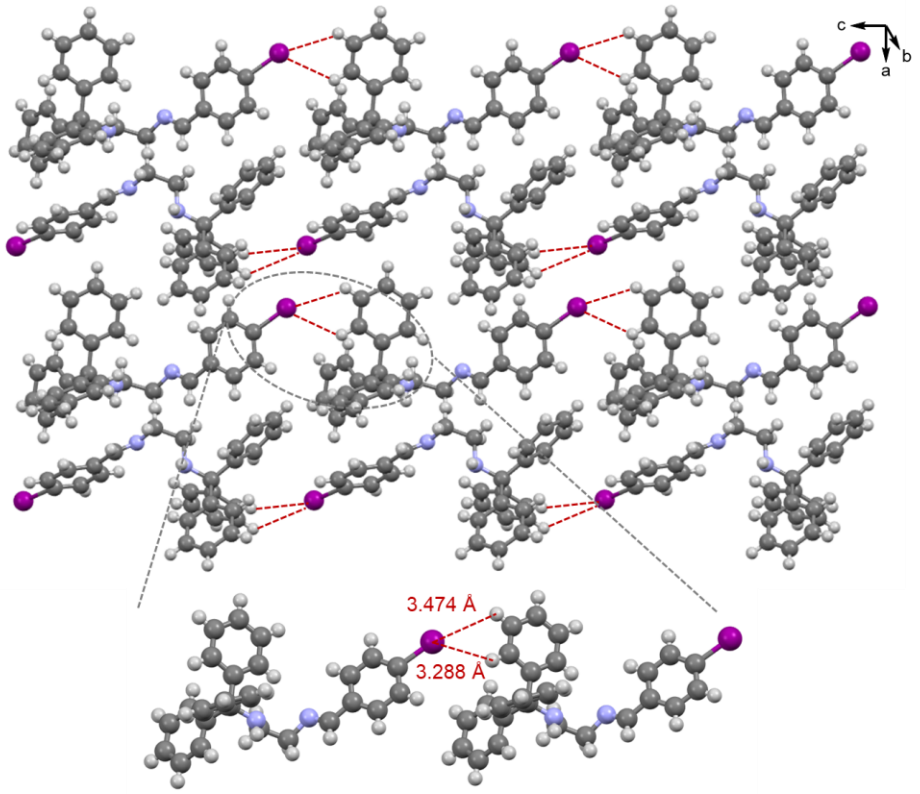

Supplementary Figure 31. Crystal packing diagram of TPMI-I.

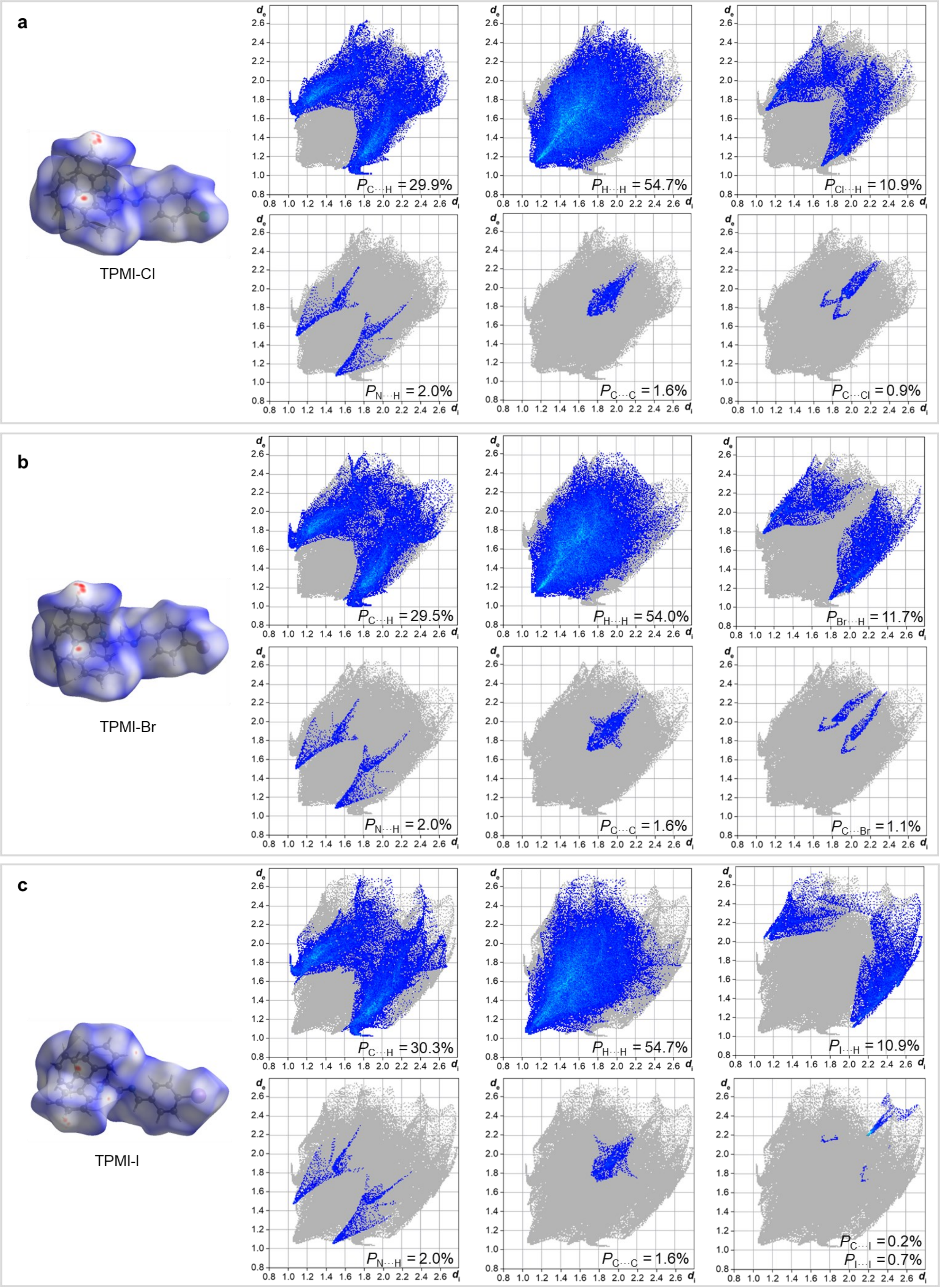

Supplementary Figure 32. Hirshfeld surfaces (mapped over d_{norm}) and decomposed fingerprint plots of **a** TPMI-Cl, **b** TPMI-Br, and **c** TPMI-I. Full fingerprints appeared as grey shadows underneath decomposed plots, and selected intermolecular interactions were shown as a blue shadow. The proportions of different intermolecular interactions to the total interactions are indicated.

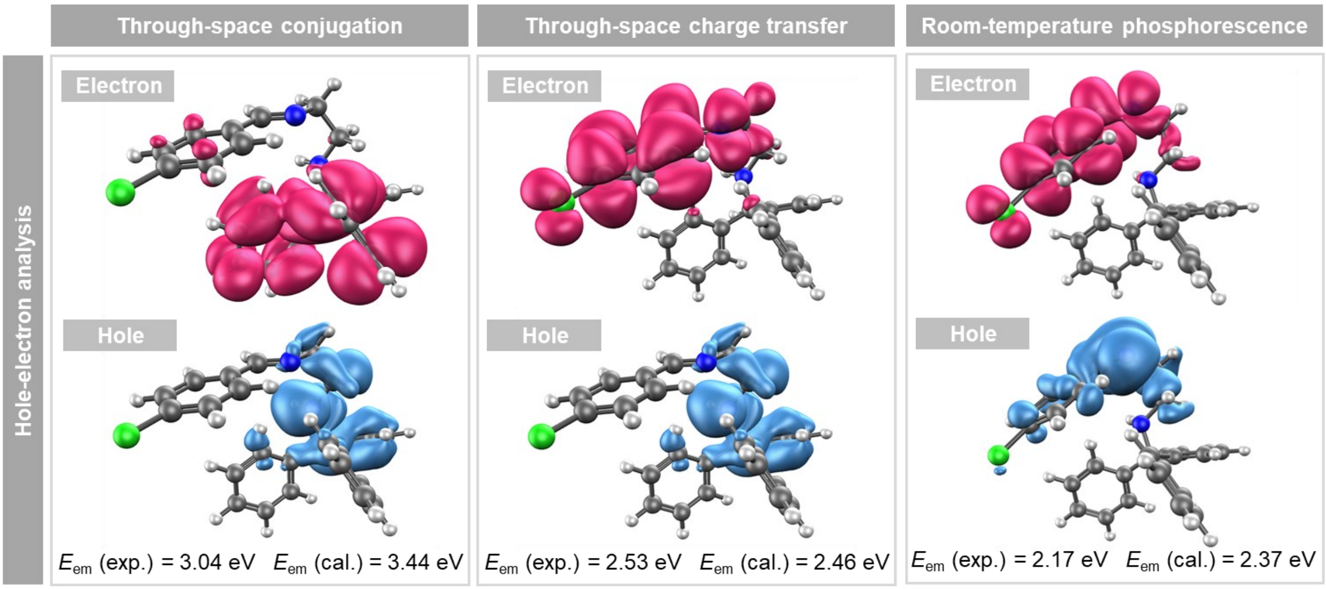

Supplementary Figure 33. Natural transition orbitals of TPMI-Cl based on the corresponding optimized geometries calculated at B3LYP-D3/Def2-SVP level.

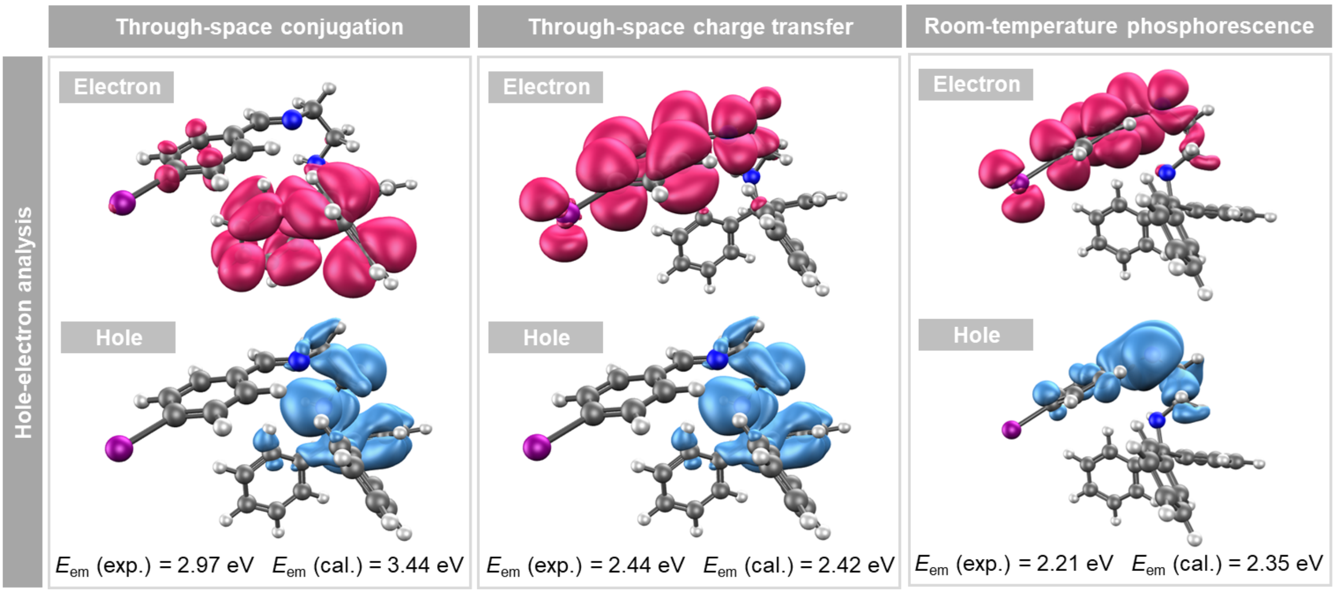

Supplementary Figure 34. Natural transition orbitals of TPMI-I based on the corresponding optimized geometries calculated at B3LYP-D3/Def2-SVP level.

Supplementary Table 2. Theoretically calculated N_1–N_2 distance and dihedral angle of $\angle N_1$-C_1-C_2-N_2 of the targeted compounds in the correspondingly optimized geometries, calculated at B3LYP-D3/Def2-SVP level.

Compound	State	N_1-N_2 Distance (Å)	Dihedral angle (°)
TPMI-Cl	Ground state	2.9360	66.23
	Excited singlet state (TSCT)	2.7922	49.66
	Excited singlet state (TSC)	2.8812	60.85
	Excited triplet state	3.0802	63.11
TPMI-Br	Ground state	2.9291	65.50
	Excited singlet state (TSCT)	2.8003	50.36
	Excited singlet state (TSC)	2.8743	60.10
	Excited triplet state	3.0807	63.17
TPMI-I	Ground state	2.9217	64.98
	Excited singlet state (TSCT)	2.8048	50.99
	Excited singlet state (TSC)	2.8830	61.25
	Excited triplet state	3.0799	63.01

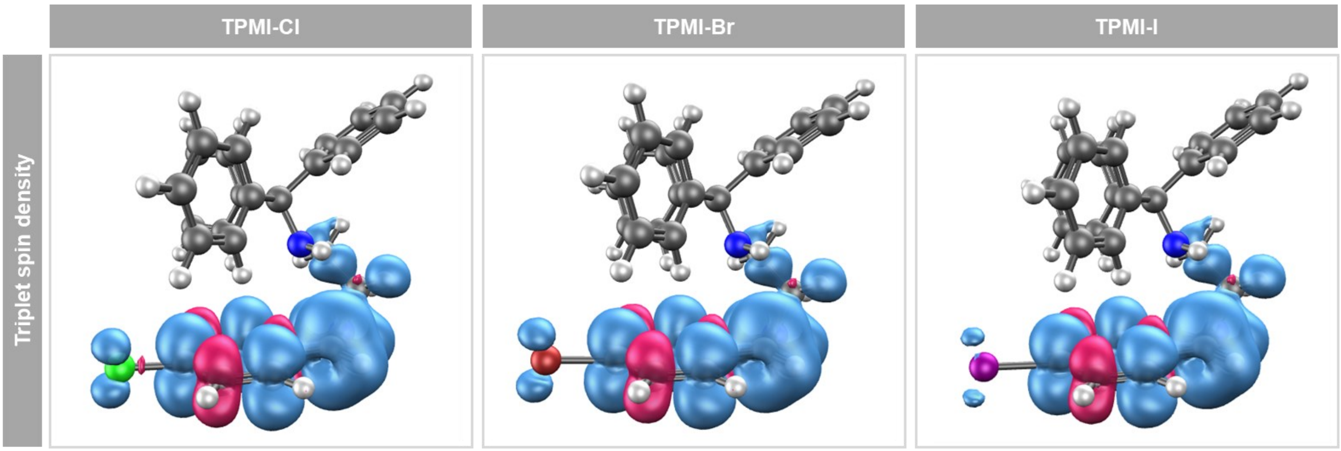

Supplementary Figure 35. Spin density maps of the triplet state of the targeted compounds based on the optimized triplet-state geometries, calculated by the UKS-DFT method, B3LYP-D3/Def2-SVP.

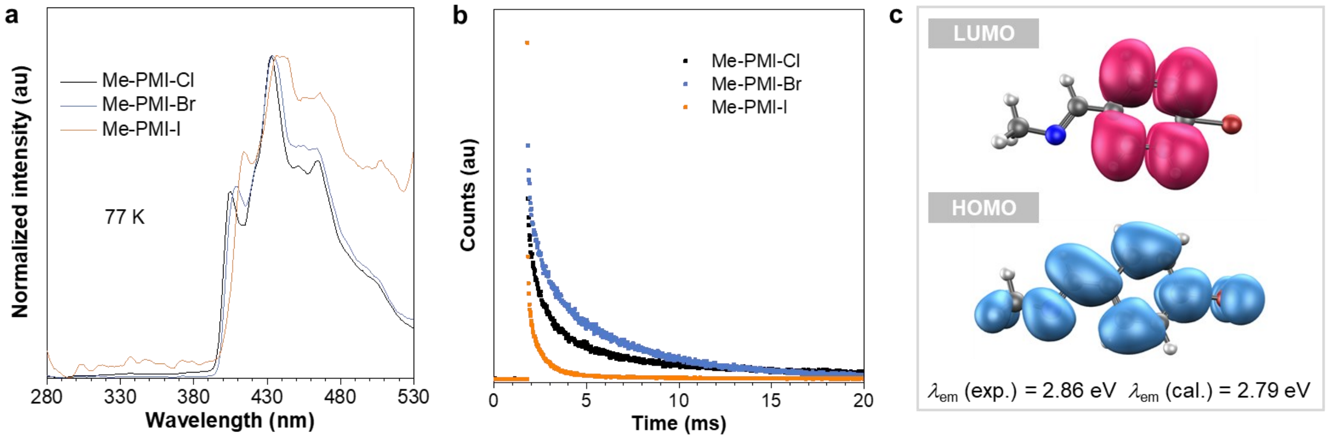

Supplementary Figure 36. a Normalized photoluminescence (PL) spectra and **b** time-resolved PL decay curves of the model compounds Me-PMI-X (X = Cl, Br, and I) in THF solution at 77 K. **c** Hole and electron analysis of Me-PMI-Br based on the optimized triplet-state geometry which corresponded to the phosphorescent transition.

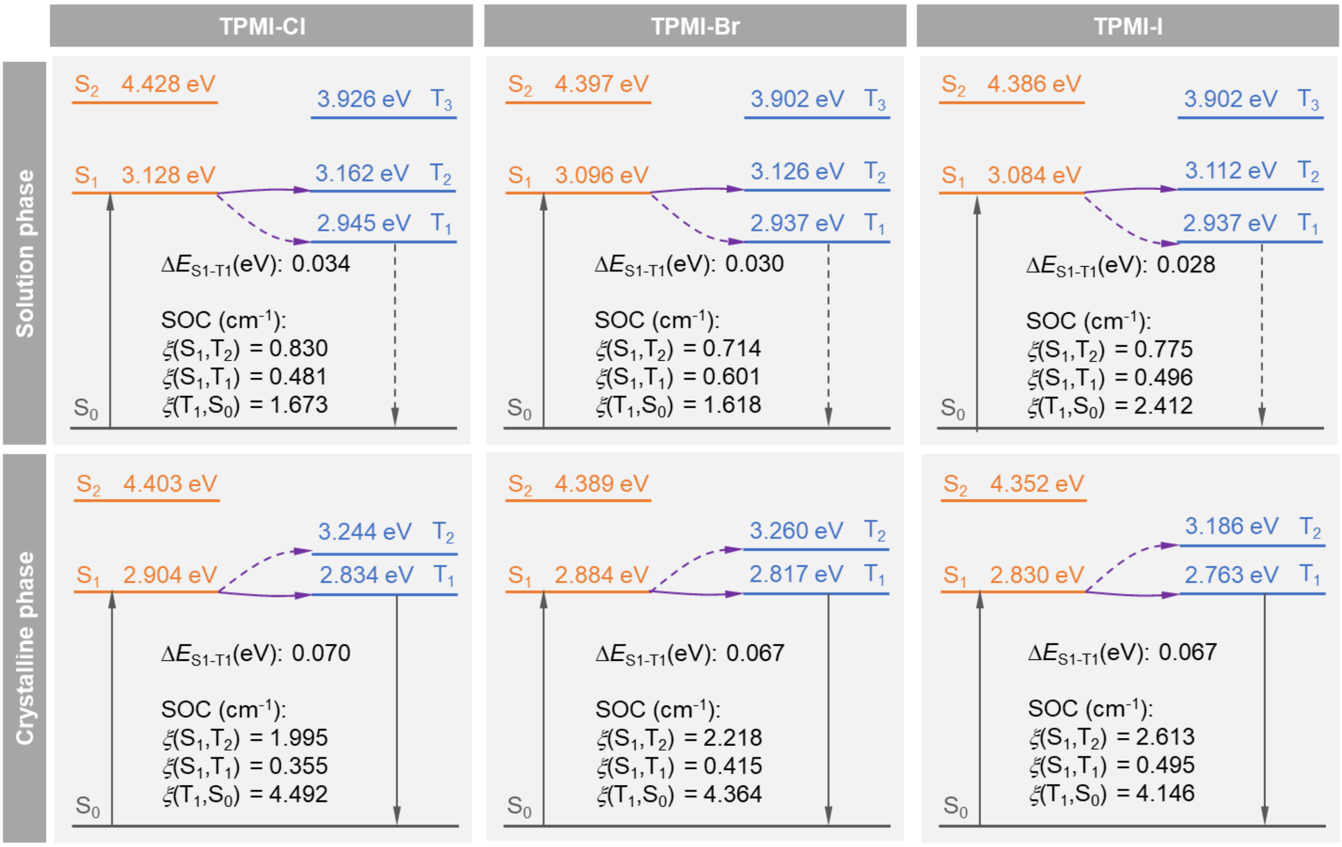

Supplementary Figure 37. Energy diagram and SOC constants (ξ) of the targeted compounds in the isolated and crystalline phases, calculated by TD-DFT method, B3LYP-D3/Def2-SVP.

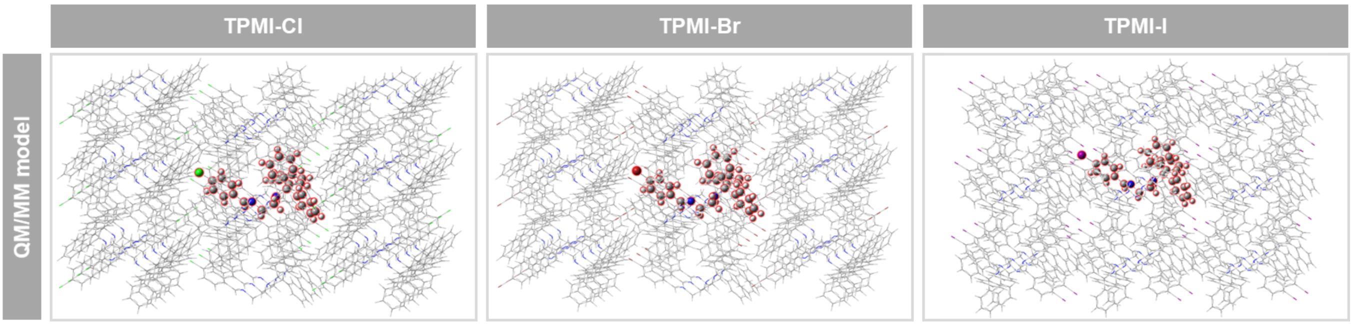

Supplementary Figure 38. Setup of QM/MM models for theoretical calculation of crystalline phase, which were extracted from crystal packing structures.

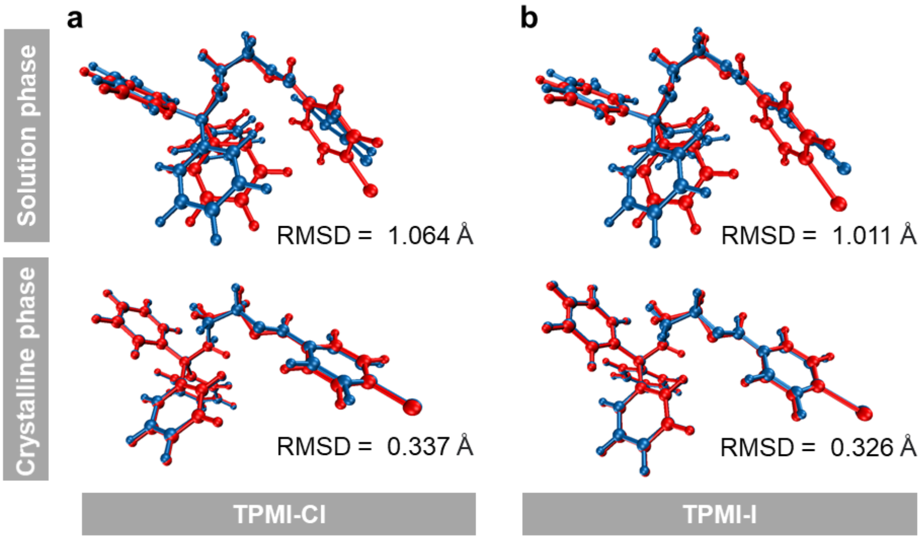

Supplementary Figure 39. Overlaps of the optimized ground-state (blue color) and singlet TSCT (red color) geometries of **a** TPMI-Cl and **b** TPMI-I in the isolated phase and crystalline phase. The root-mean-square deviation (RMSD) of atomic positions was calculated to evaluate the strength of intramolecular motions in two different phases.

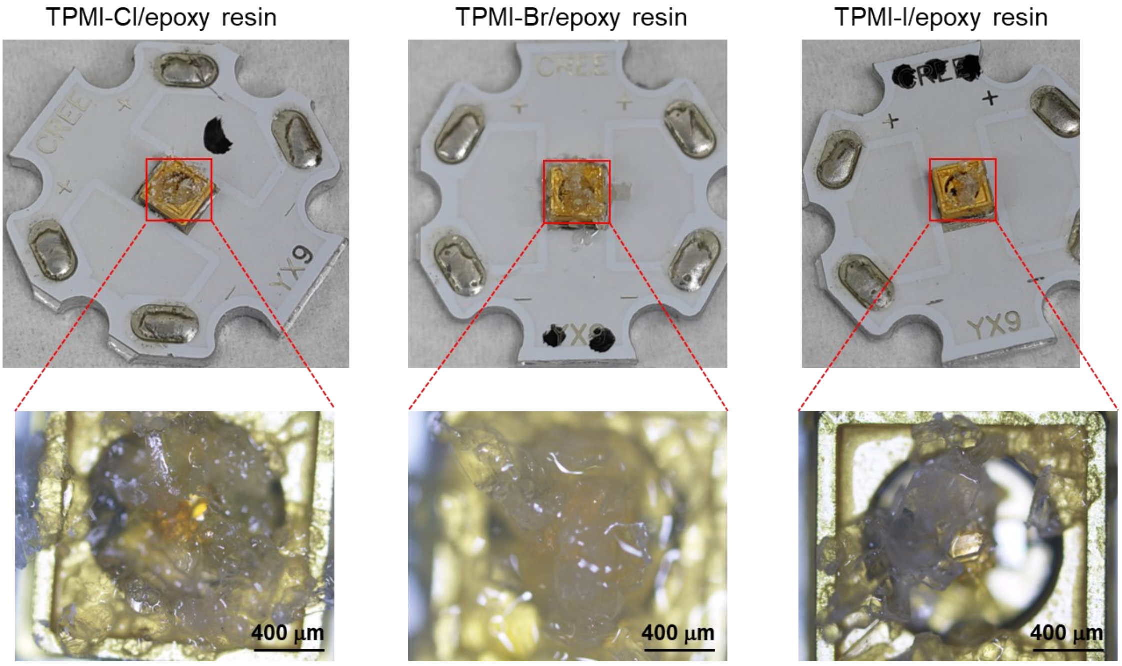

Supplementary Figure 40. Photos of the fabricated white-light emitters under the optical microscope. Microcrystals of these compounds are embedded into the epoxy resin glue.

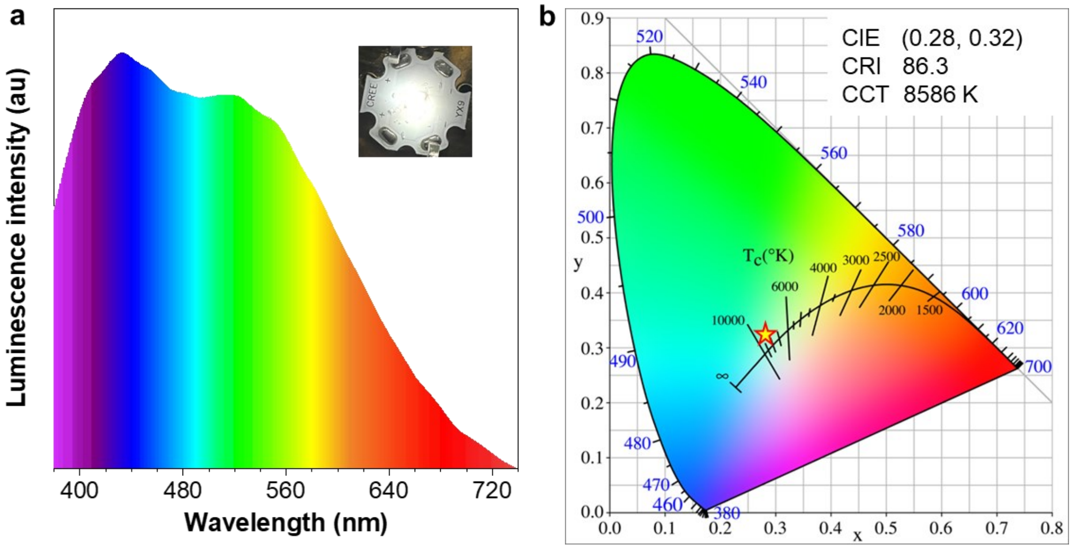

Supplementary Figure 41. a The luminescence and photo of a white-light emitter fabricated by coating a mixture of microcrystals of TPMI-Cl and epoxy resin glue on a commercial UV chip. **b** The Commission Internationale de L'Eclairage chromaticity coordinate of the fabricated emitter.

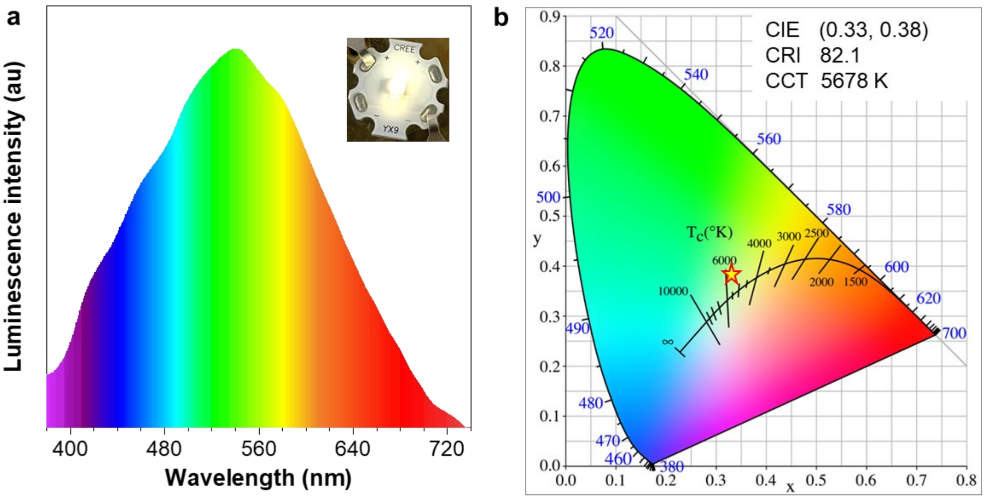

Supplementary Figure 42. a The luminescence spectra and photo of a white-light emitter fabricated by coating a mixture of microcrystals of TPMI-I and epoxy resin glue on a commercial UV chip. **b** The Commission Internationale de L'Eclairage chromaticity coordinate of the fabricated emitter.

Cartesian Coordinates of the Optimized Molecular Geometry

Supplementary Table 3. Cartesian coordinates of optimized TPMI-Cl in the ground state calculated at B3LYP-D3/Def2-SVP level.

	X	Y	Z
N	1.175129	-2.561172	-1.206440
N	-1.151785	-0.777388	-1.053130
C	-0.121408	-2.894866	-1.746710
H	-0.268056	-3.986578	-1.699149
H	-0.237529	-2.585642	-2.807446
C	-1.225335	-2.222312	-0.911042
H	-2.204515	-2.645122	-1.210561

H	-1.078751	-2.486371	0.147120
C	1.835177	-1.618068	-1.749599
H	1.491426	-1.122946	-2.680139
C	3.075232	-1.076165	-1.163937
C	3.585191	-1.569626	0.052420
H	3.056252	-2.384991	0.549284
C	4.733550	-1.023359	0.617981
H	5.128551	-1.402030	1.562132
C	5.384112	0.031651	-0.037300
C	4.898958	0.539569	-1.245488
H	5.417953	1.362270	-1.739595
C	3.745656	-0.019005	-1.800524
H	3.357843	0.378804	-2.742369
C	-1.869530	0.064426	-0.079516
C	-3.353794	-0.370873	-0.070480
C	-4.216803	0.066403	-1.091184
H	-3.869436	0.811707	-1.809962
C	-5.516040	-0.433923	-1.205820
H	-6.167200	-0.068642	-2.004407
C	-5.982582	-1.397018	-0.304223
H	-6.999796	-1.787545	-0.389575
C	-5.129307	-1.859018	0.701035
H	-5.472855	-2.621164	1.405454
C	-3.828744	-1.354439	0.811637
H	-3.176765	-1.739321	1.597209
C	-1.189902	-0.014369	1.308669
C	-1.861021	0.412035	2.466900
H	-2.903807	0.728168	2.405367
C	-1.212401	0.442027	3.704278
H	-1.757908	0.774940	4.591226
C	0.127547	0.052182	3.808253
H	0.635659	0.075350	4.775751
H	-1.438274	-0.509123	-1.993462
C	0.808763	-0.360129	2.659634
H	1.858926	-0.658887	2.717601
C	0.157015	-0.389114	1.422647
H	0.698591	-0.702825	0.532808
C	-1.683481	1.541856	-0.500748
C	-2.576454	2.536824	-0.069823
H	-3.456846	2.259481	0.512330
C	-2.358116	3.881320	-0.379775
H	-3.069973	4.636549	-0.036463
C	-1.236387	4.260636	-1.125524
H	-1.065612	5.312297	-1.369433
C	-0.334909	3.280518	-1.549361
H	0.551379	3.562160	-2.124273
C	-0.554009	1.934753	-1.236018
H	0.166065	1.178129	-1.548998
Cl	6.825611	0.725868	0.674015

Supplementary Table 4. Cartesian coordinates of optimized TPMI-Cl in the excited TSCT state calculated at B3LYP-D3/Def2-SVP level.

	X	Y	Z
N	0.756920	-2.545035	-1.292905
N	-1.361526	-0.732790	-1.136522
C	-0.447141	-2.825572	-2.017250
H	-0.703577	-3.903960	-2.023627
H	-0.405884	-2.515921	-3.087415
C	-1.667256	-2.126491	-1.367480
H	-2.543466	-2.167600	-2.039518
H	-1.951704	-2.584949	-0.413975
C	1.678816	-1.828306	-1.939713
H	1.549969	-1.617018	-3.020916
C	2.821048	-1.242283	-1.329448
C	3.103640	-1.384413	0.075886
H	2.463158	-2.033337	0.674696
C	4.148801	-0.699583	0.678322
H	4.336342	-0.809204	1.749315
C	4.972681	0.147480	-0.082510
C	4.752545	0.286712	-1.470634
H	5.405006	0.936904	-2.057708
C	3.709296	-0.395225	-2.073204
H	3.540498	-0.266863	-3.147130
C	-1.771843	0.118964	-0.014444
C	-3.218735	0.437373	-0.475752
C	-3.437846	1.443193	-1.435145
H	-2.609025	2.064332	-1.776655
C	-4.718352	1.663967	-1.945809
H	-4.879203	2.459119	-2.676713
C	-5.785959	0.865019	-1.526901
H	-6.788057	1.036861	-1.926285
C	-5.566969	-0.165701	-0.603795
H	-6.395600	-0.800562	-0.283227
C	-4.288645	-0.390256	-0.094099
H	-4.126157	-1.205416	0.611098
C	-1.652200	-0.586505	1.346455
C	-2.441948	-0.151682	2.421086
H	-3.207651	0.610815	2.266889
C	-2.250056	-0.681120	3.699509
H	-2.875331	-0.334477	4.525744
C	-1.261034	-1.645324	3.920014
H	-1.111537	-2.060197	4.919840
H	-0.942588	-0.234353	-1.922581
C	-0.460410	-2.066625	2.854539
H	0.322819	-2.811441	3.016029
C	-0.643833	-1.536509	1.573667
H	0.002223	-1.874034	0.754918
C	-0.829450	1.331734	0.059081
C	-1.274762	2.569100	0.545156

H	-2.325958	2.713396	0.801612
C	-0.373785	3.624811	0.706296
H	-0.733129	4.585929	1.081866
C	0.980303	3.454538	0.392395
H	1.680912	4.282936	0.522534
C	1.432056	2.217531	-0.074897
H	2.487258	2.052701	-0.306145
C	0.531699	1.159329	-0.231697
H	0.916864	0.192449	-0.556251
Cl	6.287182	1.039043	0.693048

Supplementary Table 5. Cartesian coordinates of optimized TPMI-Cl in the excited TSC state calculated at B3LYP-D3/Def2-SVP level.

	X	Y	Z
N	0.921906	-2.742254	0.429782
N	-1.453227	-1.338092	-0.399942
C	-0.312657	-3.435052	0.150337
H	-0.414748	-4.302579	0.822546
H	-0.377371	-3.811338	-0.892444
C	-1.509587	-2.511317	0.439820
H	-2.443971	-3.055807	0.217149
H	-1.534555	-2.189728	1.486783
C	1.735150	-2.515678	-0.524819
H	1.545219	-2.891463	-1.551391
C	2.945121	-1.693074	-0.364691
C	3.309389	-1.157427	0.885533
H	2.704080	-1.396672	1.761485
C	4.422555	-0.331769	1.006871
H	4.705608	0.088269	1.973140
C	5.180636	-0.032104	-0.134060
C	4.840023	-0.551868	-1.386214
H	5.441551	-0.305174	-2.262201
C	3.723133	-1.382581	-1.492691
H	3.447035	-1.785758	-2.470726
C	-1.887623	0.035790	-0.080176
C	-3.400389	-0.160671	-0.380721
C	-3.879230	-0.039243	-1.698362
H	-3.196956	0.276413	-2.488454
C	-5.220362	-0.296802	-1.988369
H	-5.587006	-0.179099	-3.010559
C	-6.089175	-0.708639	-0.972507
H	-7.138762	-0.911702	-1.198577
C	-5.609601	-0.873046	0.334688
H	-6.283977	-1.203959	1.127754
C	-4.270630	-0.617778	0.625846
H	-3.893495	-0.753239	1.640034
C	-1.555377	0.450077	1.342170
C	-2.318244	1.475532	1.976448
H	-3.277754	1.778620	1.551181

C	-1.861445	2.103568	3.127615
H	-2.481776	2.874797	3.594962
C	-0.618002	1.759102	3.703067
H	-0.263384	2.256040	4.608591
H	-1.305555	-1.501153	-1.397991
C	0.158907	0.769080	3.068512
H	1.130344	0.487454	3.487853
C	-0.283428	0.134432	1.914225
H	0.350703	-0.624151	1.454222
C	-1.167974	1.003549	-1.004719
C	-1.737626	2.275736	-1.313933
H	-2.789653	2.466150	-1.088408
C	-0.976314	3.276425	-1.901131
H	-1.450359	4.232978	-2.142848
C	0.393010	3.079087	-2.196670
H	0.985521	3.871899	-2.658005
C	0.977901	1.841415	-1.858471
H	2.039545	1.663446	-2.057958
C	0.227252	0.832061	-1.269048
H	0.727754	-0.096760	-0.994868
Cl	6.575807	1.011889	0.013444

Supplementary Table 6. Cartesian coordinates of optimized TPMI-Cl in the triplet state calculated at B3LYP-D3/Def2-SVP level.

	X	Y	Z
N	0.736600	-3.734185	1.099700
N	-0.620364	-1.117268	0.206290
C	-0.684227	-3.486651	1.005979
H	-1.191950	-4.095154	1.770203
H	-1.043682	-3.820968	0.011027
C	-1.138725	-2.028379	1.214338
H	-2.244979	-2.076593	1.231653
H	-0.828972	-1.694302	2.218218
C	1.585344	-3.414236	0.072525
H	1.629480	-4.151862	-0.743514
C	2.479591	-2.314646	0.070656
C	2.589307	-1.428385	1.183182
H	2.017506	-1.642136	2.087908
C	3.398851	-0.302503	1.128682
H	3.455615	0.382865	1.976176
C	4.135081	-0.032675	-0.035277
C	4.090665	-0.910418	-1.130449
H	4.680912	-0.693726	-2.022589
C	3.280846	-2.035523	-1.075514
H	3.232829	-2.705074	-1.938147
C	-1.347166	0.149694	-0.030617
C	-2.783970	-0.199967	-0.485029
C	-3.005230	-0.605864	-1.814298
H	-2.188111	-0.551580	-2.536911

C	-4.251621	-1.075885	-2.232266
H	-4.394716	-1.375380	-3.273825
C	-5.313452	-1.161846	-1.324047
H	-6.291314	-1.525590	-1.649588
C	-5.106147	-0.783335	0.003770
H	-5.920621	-0.855397	0.729299
C	-3.853873	-0.312493	0.418261
H	-3.717172	-0.029530	1.462749
C	-1.278697	1.051643	1.221959
C	-2.186048	2.106161	1.417017
H	-3.009576	2.254206	0.716741
C	-2.047650	2.983753	2.496061
H	-2.769988	3.793586	2.627769
C	-0.990467	2.831856	3.399203
H	-0.882943	3.516689	4.244197
H	-0.508449	-1.595894	-0.685582
C	-0.066781	1.801437	3.200588
H	0.776093	1.679014	3.886063
C	-0.207407	0.926541	2.119291
H	0.534660	0.149080	1.945754
C	-0.578944	0.957437	-1.105416
C	-1.218363	1.974788	-1.833859
H	-2.288683	2.145434	-1.704445
C	-0.502259	2.775304	-2.726774
H	-1.021976	3.558073	-3.285617
C	0.873010	2.580973	-2.902902
H	1.434406	3.211666	-3.597075
C	1.519506	1.577263	-2.177285
H	2.595742	1.421361	-2.284711
C	0.798941	0.772668	-1.287841
H	1.312735	0.005558	-0.714021
Cl	5.098753	1.427972	-0.138428

Supplementary Table 7. Cartesian coordinates of optimized TPMI-Br in the ground state calculated at B3LYP-D3/Def2-SVP level.

	X	Y	Z
Br	6.466128	0.532376	0.432755
N	0.511642	-2.726271	-1.110539
N	-1.731940	-0.846304	-1.001935
C	-0.811394	-3.043843	-1.594173
H	-0.997497	-4.123576	-1.471807
H	-0.944934	-2.799178	-2.669532
C	-1.865714	-2.276061	-0.776449
H	-2.868745	-2.671068	-1.031629
H	-1.706533	-2.486354	0.291838
C	1.184325	-1.840743	-1.729689
H	0.827078	-1.391665	-2.678238
C	2.460010	-1.306900	-1.217729
C	2.998565	-1.746644	0.006450

H	2.464440	-2.515324	0.568073
C	4.184158	-1.207519	0.498970
H	4.596752	-1.549670	1.449625
C	4.841888	-0.213915	-0.239627
C	4.328593	0.241081	-1.456959
H	4.848695	1.016858	-2.020842
C	3.138493	-0.310471	-1.937863
H	2.728716	0.045593	-2.887163
C	-2.392801	0.080273	-0.066312
C	-3.896734	-0.277954	-0.020243
C	-4.747444	0.148430	-1.055910
H	-4.369820	0.835133	-1.816613
C	-6.071641	-0.289493	-1.133178
H	-6.711766	0.065899	-1.945031
C	-6.576963	-1.177994	-0.177485
H	-7.613590	-1.519704	-0.233637
C	-5.737625	-1.629112	0.844308
H	-6.112137	-2.333767	1.591513
C	-4.411809	-1.187046	0.917333
H	-3.771918	-1.562108	1.717310
C	-1.698221	0.039476	1.316116
C	-2.331611	0.555600	2.458975
H	-3.359431	0.916803	2.394288
C	-1.663873	0.618839	3.684890
H	-2.180272	1.021827	4.560102
C	-0.342035	0.172497	3.792261
H	0.181045	0.221560	4.750770
H	-2.022803	-0.617656	-1.951325
C	0.302171	-0.330119	2.658277
H	1.338186	-0.674234	2.719174
C	-0.368351	-0.392023	1.432621
H	0.145379	-0.776177	0.553794
C	-2.139583	1.522970	-0.566032
C	-2.975867	2.581828	-0.175337
H	-3.860764	2.379031	0.430396
C	-2.696093	3.896642	-0.555227
H	-3.364761	4.702521	-0.241496
C	-1.568167	4.182017	-1.332737
H	-1.349060	5.210413	-1.631079
C	-0.723163	3.137907	-1.718232
H	0.167203	3.345414	-2.317856
C	-1.003956	1.821914	-1.335162
H	-0.326821	1.016473	-1.620895

Supplementary Table 8. Cartesian coordinates of optimized TPMI-Br in the excited TSCT state calculated at B3LYP-D3/Def2-SVP level.

	X	Y	Z
Br	5.999993	0.812975	0.324222
N	0.098418	-2.866952	-0.731458

N	-1.943639	-0.963259	-0.950171
C	-1.138718	-3.241470	-1.349308
H	-1.441300	-4.280646	-1.109788
H	-1.118122	-3.178964	-2.462180
C	-2.307688	-2.358197	-0.844759
H	-3.207299	-2.515180	-1.466587
H	-2.573596	-2.573795	0.195781
C	1.037571	-2.376500	-1.542049
H	0.889336	-2.414574	-2.640518
C	2.226419	-1.732502	-1.103108
C	2.539110	-1.558704	0.291947
H	1.879919	-2.009033	1.035234
C	3.638763	-0.820392	0.703261
H	3.843723	-0.691847	1.768934
C	4.489320	-0.229041	-0.247799
C	4.238321	-0.403112	-1.627163
H	4.906880	0.046573	-2.364626
C	3.139898	-1.139361	-2.037440
H	2.949433	-1.255468	-3.109214
C	-2.262129	0.139828	-0.036346
C	-3.709628	0.429845	-0.514921
C	-3.924816	1.233729	-1.649001
H	-3.084556	1.733414	-2.132001
C	-5.214743	1.409359	-2.153528
H	-5.372462	2.050185	-3.023400
C	-6.296449	0.761754	-1.549772
H	-7.305800	0.898662	-1.944292
C	-6.083427	-0.074750	-0.446406
H	-6.923794	-0.593137	0.019850
C	-4.796028	-0.254163	0.057836
H	-4.636741	-0.919445	0.906449
C	-2.124602	-0.248661	1.444618
C	-2.859885	0.449471	2.413901
H	-3.602581	1.190622	2.112794
C	-2.643578	0.210898	3.773340
H	-3.226141	0.761034	4.516148
C	-1.685513	-0.723630	4.179631
H	-1.517422	-0.911226	5.242948
H	-1.559988	-0.673483	-1.850532
C	-0.940232	-1.408837	3.215868
H	-0.182173	-2.134665	3.520483
C	-1.147239	-1.169577	1.853821
H	-0.544888	-1.711303	1.115087
C	-1.258712	1.281865	-0.266653
C	-1.614907	2.616646	-0.025877
H	-2.641039	2.871651	0.245870
C	-0.657403	3.628388	-0.130854
H	-0.947832	4.665146	0.055357
C	0.665982	3.318845	-0.468813
H	1.411776	4.113655	-0.546573

C	1.029500	1.988401	-0.692098
H	2.060135	1.718137	-0.934516
C	0.072398	0.974797	-0.582995
H	0.391815	-0.058530	-0.719067

Supplementary Table 9. Cartesian coordinates of optimized TPMI-Br in the excited TSC state calculated at B3LYP-D3/Def2-SVP level.

	X	Y	Z
Br	6.252930	0.720573	-0.048873
N	0.283462	-2.799837	0.601595
N	-2.020098	-1.350066	-0.322151
C	-0.976554	-3.459472	0.355582
H	-1.117360	-4.278450	1.079881
H	-1.048364	-3.897475	-0.662297
C	-2.137642	-2.473932	0.576866
H	-3.091086	-2.990582	0.368411
H	-2.163414	-2.096871	1.604959
C	1.120944	-2.695097	-0.353074
H	0.927368	-3.149543	-1.346492
C	2.368250	-1.921068	-0.244015
C	2.742954	-1.291730	0.958223
H	2.115567	-1.418336	1.842184
C	3.894886	-0.512785	1.022977
H	4.180583	-0.022968	1.955105
C	4.680468	-0.356560	-0.127864
C	4.331092	-0.972417	-1.332796
H	4.950878	-0.841502	-2.220856
C	3.174648	-1.753827	-1.381950
H	2.891175	-2.231422	-2.323736
C	-2.399904	0.055955	-0.085360
C	-3.909251	-0.085755	-0.430744
C	-4.342885	0.012371	-1.765770
H	-3.624863	0.271360	-2.544639
C	-5.685231	-0.195718	-2.088109
H	-6.016319	-0.095643	-3.124234
C	-6.601184	-0.535365	-1.086969
H	-7.651623	-0.699678	-1.339051
C	-6.168372	-0.676808	0.238959
H	-6.879766	-0.951496	1.021038
C	-4.828408	-0.470173	0.562461
H	-4.487285	-0.586199	1.591695
C	-2.098919	0.526190	1.326297
C	-2.838605	1.612545	1.882276
H	-3.768692	1.933884	1.407746
C	-2.395739	2.276834	3.018431
H	-2.998094	3.095276	3.425102
C	-1.189788	1.909526	3.655800
H	-0.846397	2.434482	4.549754
H	-1.858217	-1.571752	-1.306459

C	-0.433465	0.859111	3.097170
H	0.509847	0.559020	3.565082
C	-0.861323	0.188257	1.958170
H	-0.243037	-0.614890	1.555240
C	-1.604941	0.944127	-1.028191
C	-2.098552	2.230986	-1.401241
H	-3.144371	2.484446	-1.211839
C	-1.270323	3.167561	-2.003390
H	-1.686552	4.137549	-2.293223
C	0.093836	2.888409	-2.252504
H	0.739332	3.631184	-2.725876
C	0.603337	1.635964	-1.852761
H	1.658787	1.395412	-2.016234
C	-0.214822	0.690412	-1.247134
H	0.228766	-0.251773	-0.924846

Supplementary Table 10. Cartesian coordinates of optimized TPMI-Br in the triplet state calculated at B3LYP-D3/Def2-SVP level.

	X	Y	Z
Br	4.916045	0.937088	-0.068264
N	-0.061778	-3.890509	1.036658
N	-1.149042	-1.138136	0.180348
C	-1.449371	-3.495611	0.956753
H	-2.013001	-4.058775	1.716439
H	-1.845988	-3.779084	-0.039944
C	-1.751126	-2.000568	1.184168
H	-2.856478	-1.935612	1.211208
H	-1.400339	-1.710356	2.188039
C	0.817585	-3.637527	0.017575
H	0.786244	-4.356043	-0.815895
C	1.822951	-2.639216	0.043703
C	2.016278	-1.794801	1.176857
H	1.419382	-1.968855	2.073973
C	2.937356	-0.757043	1.152490
H	3.053894	-0.105150	2.020110
C	3.706413	-0.538845	-0.001340
C	3.580314	-1.383291	-1.116600
H	4.193998	-1.215237	-2.003476
C	2.657721	-2.419906	-1.091312
H	2.547625	-3.061446	-1.969429
C	-1.731782	0.206006	-0.030371
C	-3.203193	0.023189	-0.471616
C	-3.482058	-0.336596	-1.803310
H	-2.672319	-0.358791	-2.535876
C	-4.776335	-0.664881	-2.210811
H	-4.963051	-0.931769	-3.254354
C	-5.830313	-0.651534	-1.289546
H	-6.844970	-0.904972	-1.606924
C	-5.568265	-0.316851	0.040397

H	-6.377097	-0.313034	0.775714
C	-4.268164	0.012262	0.444281
H	-4.088837	0.263564	1.490370
C	-1.548760	1.077781	1.232245
C	-2.334995	2.219640	1.457586
H	-3.149073	2.464350	0.773734
C	-2.085284	3.060796	2.545767
H	-2.714706	3.940843	2.701613
C	-1.035089	2.783303	3.427010
H	-0.840896	3.439955	4.278872
H	-1.108868	-1.612998	-0.719662
C	-0.229956	1.663623	3.197454
H	0.606626	1.441990	3.865509
C	-0.481624	0.825696	2.107313
H	0.169351	-0.023735	1.908226
C	-0.889034	0.934778	-1.105710
C	-1.413931	2.032729	-1.807975
H	-2.453481	2.328343	-1.654684
C	-0.622816	2.754407	-2.704758
H	-1.053928	3.602981	-3.242506
C	0.714588	2.396248	-2.913109
H	1.334863	2.963341	-3.612012
C	1.247750	1.310602	-2.213822
H	2.293030	1.022264	-2.350114
C	0.452821	0.587773	-1.317716
H	0.882087	-0.242314	-0.762318

Supplementary Table 11. Cartesian coordinates of optimized TPMI-I in the ground state calculated at B3LYP-D3/Def2-SVP level.

	X	Y	Z
N	-0.091155	-2.862118	-1.027963
N	-2.256293	-0.901507	-0.963065
C	-1.434829	-3.159609	-1.465481
H	-1.654830	-4.224070	-1.281612
H	-1.585383	-2.966693	-2.548992
C	-2.442144	-2.312134	-0.666622
H	-3.464978	-2.678058	-0.884007
H	-2.269861	-2.477305	0.407356
C	0.591875	-2.028405	-1.705111
H	0.226492	-1.617364	-2.667737
C	1.889917	-1.501462	-1.244609
C	2.446081	-1.898228	-0.014119
H	1.911431	-2.633539	0.590256
C	3.649265	-1.357025	0.433369
H	4.067528	-1.670732	1.391469
C	4.308185	-0.404099	-0.357341
C	3.776929	0.005109	-1.584031
H	4.290420	0.748440	-2.195893
C	2.569614	-0.548356	-2.019545

H	2.146784	-0.225799	-2.975285
C	-2.857102	0.095291	-0.060245
C	-4.374253	-0.192727	0.029294
C	-5.226343	0.225435	-1.008631
H	-4.834177	0.859195	-1.806997
C	-6.570161	-0.154417	-1.040985
H	-7.210517	0.193447	-1.855906
C	-7.094700	-0.975559	-0.036627
H	-8.146550	-1.271698	-0.057725
C	-6.255286	-1.418883	0.988495
H	-6.645396	-2.071810	1.773731
C	-4.909727	-1.035064	1.016499
H	-4.270951	-1.403059	1.820705
C	-2.138460	0.087935	1.310725
C	-2.726885	0.685494	2.437785
H	-3.738473	1.089877	2.372695
C	-2.034223	0.776119	3.647996
H	-2.515803	1.242705	4.511396
C	-0.731644	0.276243	3.754737
H	-0.189138	0.346714	4.701041
H	-2.556146	-0.705548	-1.916911
C	-0.131271	-0.307169	2.635476
H	0.889739	-0.693664	2.695382
C	-0.826836	-0.396625	1.425548
H	-0.347173	-0.844805	0.557920
C	-2.550519	1.501055	-0.630480
C	-3.332125	2.612499	-0.274237
H	-4.213691	2.476114	0.354336
C	-3.001558	3.895493	-0.716710
H	-3.628229	4.743584	-0.428553
C	-1.876111	4.095497	-1.523833
H	-1.616917	5.099000	-1.870849
C	-1.085014	2.998562	-1.875825
H	-0.196908	3.139594	-2.497681
C	-1.416738	1.714440	-1.430302
H	-0.781002	0.867570	-1.690159
I	6.140948	0.440506	0.326190

Supplementary Table 12. Cartesian coordinates of optimized TPMI-I in the excited TSCT state calculated at B3LYP-D3/Def2-SVP level.

	X	Y	Z
N	-0.476120	-2.992959	-0.434383
N	-2.459107	-1.050297	-0.835075
C	-1.735254	-3.383596	-0.994697
H	-2.070138	-4.381455	-0.646743
H	-1.729548	-3.433719	-2.108350
C	-2.864260	-2.411710	-0.568489
H	-3.781350	-2.607058	-1.152752
H	-3.112447	-2.503251	0.494360

C	0.470951	-2.639626	-1.304027
H	0.304279	-2.795092	-2.389335
C	1.691507	-2.000841	-0.953132
C	2.033068	-1.684057	0.409155
H	1.370136	-2.021328	1.207302
C	3.164815	-0.945766	0.722324
H	3.383576	-0.710728	1.766988
C	4.022450	-0.495708	-0.299106
C	3.743840	-0.818439	-1.647390
H	4.412000	-0.485313	-2.444872
C	2.612905	-1.554856	-1.958242
H	2.402157	-1.784101	-3.007881
C	-2.714980	0.158397	-0.042330
C	-4.154741	0.458993	-0.537518
C	-4.347806	1.140253	-1.753146
H	-3.491906	1.543274	-2.295384
C	-5.634396	1.318198	-2.265119
H	-5.773376	1.864781	-3.200080
C	-6.736848	0.792355	-1.585252
H	-7.743726	0.930600	-1.985569
C	-6.548107	0.075310	-0.396763
H	-7.405169	-0.348420	0.130701
C	-5.264125	-0.105798	0.115782
H	-5.126812	-0.677362	1.033831
C	-2.571390	-0.071817	1.470598
C	-3.266488	0.755186	2.365391
H	-3.984998	1.487458	1.992562
C	-3.038981	0.658352	3.740465
H	-3.590252	1.307644	4.424787
C	-2.109034	-0.261099	4.236351
H	-1.932240	-0.337646	5.311980
H	-2.092451	-0.874580	-1.771373
C	-1.402213	-1.074026	3.345578
H	-0.665579	-1.789282	3.719787
C	-1.620574	-0.977399	1.967960
H	-1.047697	-1.617547	1.286930
C	-1.665404	1.221692	-0.405927
C	-1.958276	2.590079	-0.317678
H	-2.968874	2.921830	-0.072192
C	-0.956221	3.537571	-0.541304
H	-1.197043	4.601207	-0.473752
C	0.348631	3.130327	-0.846420
H	1.129289	3.875599	-1.017151
C	0.649308	1.767717	-0.916940
H	1.664454	1.423787	-1.129571
C	-0.352407	0.819273	-0.689192
H	-0.083014	-0.236562	-0.706346
I	5.752858	0.659552	0.185146

Supplementary Table 13. Cartesian coordinates of optimized TPMI-I in the excited TSC state calculated at B3LYP-D3/Def2-SVP level.

	X	Y	Z
N	0.280347	2.877880	0.518648
N	2.554583	1.333401	-0.349827
C	1.564357	3.485836	0.266572
H	1.724695	4.321839	0.966924
H	1.662519	3.888942	-0.763560
C	2.687513	2.468116	0.533155
H	3.662180	2.949359	0.339209
H	2.675445	2.109344	1.568068
C	-0.551897	2.766551	-0.439654
H	-0.335494	3.177246	-1.447314
C	-1.826128	2.040242	-0.308646
C	-2.222203	1.466929	0.914127
H	-1.589436	1.600429	1.793369
C	-3.403178	0.734637	1.006275
H	-3.697167	0.291775	1.959024
C	-4.198997	0.568532	-0.136848
C	-3.826237	1.129348	-1.362376
H	-4.446434	0.997247	-2.250165
C	-2.640071	1.864341	-1.439387
H	-2.341308	2.298694	-2.397397
C	2.878320	-0.080931	-0.081507
C	4.402249	0.006569	-0.377903
C	4.874008	-0.111364	-1.698378
H	4.172230	-0.350734	-2.498109
C	6.231607	0.052840	-1.979497
H	6.591276	-0.061396	-3.004567
C	7.126325	0.367105	-0.951225
H	8.188756	0.497356	-1.170838
C	6.657178	0.527019	0.360235
H	7.352428	0.781673	1.163349
C	5.301907	0.364714	0.642510
H	4.932682	0.496233	1.660111
C	2.514209	-0.520916	1.324994
C	3.192723	-1.627889	1.917486
H	4.125450	-1.989623	1.478539
C	2.685857	-2.262005	3.044150
H	3.242107	-3.098123	3.479547
C	1.473570	-1.842293	3.635410
H	1.079629	-2.344368	4.521624
H	2.431061	1.544971	-1.341793
C	0.778177	-0.769657	3.041084
H	-0.168356	-0.428530	3.473042
C	1.270609	-0.128298	1.911345
H	0.697921	0.693693	1.480589
C	2.085845	-0.954825	-1.039864
C	2.550190	-2.261042	-1.381829

H	3.581220	-2.544137	-1.156545
C	1.712588	-3.179274	-1.998972
H	2.107345	-4.165202	-2.264029
C	0.366511	-2.861119	-2.295351
H	-0.286761	-3.589547	-2.780180
C	-0.115524	-1.588109	-1.927679
H	-1.157011	-1.317124	-2.128732
C	0.712032	-0.660549	-1.307336
H	0.288081	0.299680	-1.011985
I	-6.000506	-0.556712	-0.001420

Supplementary Table 14. Cartesian coordinates of optimized TPMI-I in the triplet state calculated at B3LYP-D3/Def2-SVP level

	X	Y	Z
N	0.696906	3.962968	0.999145
N	1.602159	1.138318	0.169685
C	2.054582	3.472526	0.938857
H	2.647158	4.001295	1.701126
H	2.480034	3.723077	-0.054868
C	2.252027	1.961786	1.175968
H	3.350309	1.822419	1.213713
H	1.872708	1.700813	2.177485
C	-0.194523	3.750253	-0.018045
H	-0.112204	4.448842	-0.864879
C	-1.266731	2.824623	0.023457
C	-1.516976	2.013332	1.169062
H	-0.909711	2.159839	2.064319
C	-2.506959	1.040241	1.160745
H	-2.660900	0.416843	2.043455
C	-3.292111	0.854622	0.010707
C	-3.106479	1.672356	-1.117639
H	-3.725523	1.539165	-2.006777
C	-2.115055	2.644456	-1.107911
H	-1.962009	3.262484	-1.996567
C	2.090703	-0.244711	-0.029093
C	3.575451	-0.169175	-0.456883
C	3.891182	0.158507	-1.788528
H	3.091709	0.229684	-2.529050
C	5.208877	0.393010	-2.185760
H	5.423478	0.637213	-3.229500
C	6.250564	0.314906	-1.253785
H	7.283293	0.495094	-1.562806
C	5.953474	0.009643	0.075905
H	6.753238	-0.044505	0.819121
C	4.629937	-0.225340	0.469446
H	4.423924	-0.455611	1.515492
C	1.834653	-1.094787	1.235209
C	2.538228	-2.286313	1.476463
H	3.341666	-2.591143	0.804212

C	2.219122	-3.101285	2.566531
H	2.785230	-4.021140	2.734837
C	1.180396	-2.747057	3.433681
H	0.932051	-3.383414	4.286882
H	1.605405	1.611044	-0.732383
C	0.456486	-1.576524	3.188110
H	-0.370366	-1.293680	3.845113
C	0.777522	-0.764918	2.096343
H	0.188158	0.125465	1.885587
C	1.209015	-0.916502	-1.110281
C	1.657643	-2.057765	-1.796076
H	2.668325	-2.432233	-1.623062
C	0.826957	-2.722470	-2.700975
H	1.198519	-3.606871	-3.225266
C	-0.474223	-2.261344	-2.935265
H	-1.125298	-2.782901	-3.641562
C	-0.931976	-1.132089	-2.251926
H	-1.948191	-0.762229	-2.409681
C	-0.098206	-0.467693	-1.345920
H	-0.470289	0.396870	-0.801844
I	-4.744824	-0.697342	-0.040175
